# Differential responses to immune checkpoint inhibitor dictated by pre-existing differential immune profiles in squamous cell carcinomas caused by same initial oncogenic drivers

**DOI:** 10.1186/s13046-022-02337-x

**Published:** 2022-04-02

**Authors:** Samantha M. Y. Chen, Vince Popolizio, Rachel A. Woolaver, Huaibin Ge, Alexandra L. Krinsky, Jessy John, Etienne Danis, Yao Ke, Yonatan Kramer, Li Bian, Andrew G. Nicklawsky, Dexiang Gao, Silvia Liu, Zhangguo Chen, Xiao-jing Wang, Jing H. Wang

**Affiliations:** 1 Department of Immunology and Microbiology, University of Colorado Anschutz Medical Campus, School of Medicine, Aurora, CO 80045 USA; 2 Department of Pathology, University of Colorado Anschutz Medical Campus, School of Medicine, Aurora, CO 80045 USA; 3 UPMC Hillman Cancer Center, Division of Hematology and Oncology, Department of Medicine, University of Pittsburgh, Pittsburgh, PA 15213 USA; 4 Department of Pharmacology, University of Colorado Anschutz Medical Campus, School of Medicine, Aurora, CO 80045 USA; 5 Department of Pediatrics and Department of Biostatistics and Informatics, Cancer Center Biostatistics Core, University of Colorado Anschutz Medical Campus, School of Medicine, Aurora, CO 80045 USA; 6 Department of Pathology, School of Medicine, University of Pittsburgh, Pittsburgh, PA 15213 USA

**Keywords:** Cancer immunotherapy, Head and neck cancers, Immune tumor microenvironment, Tumor infiltrating lymphocytes, p53 mutations, PIK3CA hyperactivation

## Abstract

**Background:**

While immune checkpoint inhibitors (ICI) were approved for head and neck squamous cell carcinomas (HNSCCs), the response rate remains relatively low. Mechanisms underlying ICI unresponsiveness versus sensitivity are not fully understood.

**Method:**

To better delineate differential responses to ICI treatment, we employed mouse SCC models, termed KPPA tumors that were caused by deleting *p53* and hyperactivating *PIK3CA*, two most frequently mutated genes in human HNSCCs. We transplanted two KPPA tumor lines (TAb2 versus TCh3) into C57BL/6 recipients and examined the immune tumor microenvironment using flow cytometry. Furthermore, we employed single-cell RNA sequencing to identify the difference in tumor infiltrating lymphocytes (TILs).

**Results:**

We found that different KPPA tumors exhibited heterogeneous immune profiles pre-existing treatment that dictated their sensitivity or unresponsiveness to anti-PD-L1. Unresponsive TAb2 tumors were highly enriched with functional tumor-associated macrophages (TAMs), especially M2-TAMs. In contrast, sensitive TCh3 tumors contained more CD8 TILs with better effector functions. TAb2 tumor cells drastically expanded F4/80^+^ TAMs from bone marrow precursors, requiring CSF1 and VEGF. Consistently, a higher combined expression of VEGF-C and CSF1 predicts worse survival in PIK3CA^Amp^/TP53^Mutated^ HNSCC patients. Unresponsive TAb2 tumors upregulated distinct signaling pathways that correlate with aggressive tumor phenotypes. While anti-PD-L1 did not affect the TME of TAb2 tumors, it significantly increased the number of CD8 TILs in TCh3 tumors.

**Conclusions:**

We uncovered tumor-intrinsic differences that may underlie the differential responses to ICI by establishing and employing two SCC tumor lines, TAb2 vs. TCh3, both of which harbor *TP53* deletion and *PIK3CA* hyperactivation. Our study indicates the limitation of stratifying cancers according to their genetic alterations and suggests that evaluating HNSCC tumor-intrinsic cues along with immune profiles in the TME may help better predict ICI responses. Our experimental models may provide a platform for pinpointing tumor-intrinsic differences underlying an immunosuppressive TME in HNSCCs and for testing combined immunotherapies targeting either tumor-specific or TAM-specific players to improve ICI efficacy.

**Supplementary Information:**

The online version contains supplementary material available at 10.1186/s13046-022-02337-x.

## Background

About 90% of head and neck cancers (HNCs) constitute head and neck squamous cell carcinomas (HNSCCs). HNSCCs result in a high morbidity and mortality rate with only 50–60% of patients having a 5-year survival rate [1]. HNSCCs are often associated with carcinogens, such as alcohol and tobacco use, or oncogenic human papillomavirus (HPV) infection [2, 3], thus, are categorized as HPV^−^ or HPV^+^ HNSCCs. HNSCCs display a high rate of genetic heterogeneity, consisting of hyper-activation of oncogenes (e.g., *PIK3CA and HRAS*) or both loss-of-function mutations and potential gain-of-function mutations in multiple genes (e.g., *TP53*, *CASP8* and *CREBBP/EP300*) [4–14]. Phosphoinositide 3-kinase (PI3K) is a frequently dysregulated pathway in HNSCCs with a *PIK3CA* gene mutation rate of approximately 16% and gene amplification rate of more than 30% [4, 15]. However, therapies targeting the PI3K pathway have had limited efficacy in HNSCCs so far [16]. Another highly mutated gene in HNSCCs is the *TP53* tumor suppressor gene, with over 80% of HPV^−^ HNSCCs harboring *TP53* mutations, whereas *TP53* mutations occur much less frequently in HPV^+^ HNSCCs (~ 3%) [3, 4]. While clinical trials have tested several therapies targeting p53, they have yet to be proven effective [17–20]. In general, *TP53* mutations in HNSCC are associated with poor prognosis and overall survival with increased rate of recurrence and resistance to therapies [7–9, 20, 21]. Thus, it would be of great interest to better understand how these two genetic alterations influence the aggressive phenotypes of HNSCCs, thereby laying a scientific foundation for developing more effective therapies.

Our prior studies showed that HNSCC patients with *PIK3CA* amplification (PIK3CA^Amp^) exhibited a higher frequency of harboring *TP53* mutations (TP53^Mutated^) compared with patients with WT *PIK3CA* [22]. In addition, we found that HNSCC patients with dual genetic alterations, i.e., PIK3CA^Amp^/TP53^Mutated^, showed a significantly worse prognosis in their 10-year overall survival than PIK3CA^WT^/TP53^WT^ group [22]. However, the underlying mechanisms that lead to worse outcomes in PIK3CA^Amp^/TP53^Mutated^ HNSCC patients remain incompletely understood. In this regard, prior studies have generated murine models that mimicked the alterations of *PIK3CA*, *p53* or both in HNSCCs [23–25]; however, none of the prior studies showed that genetic alterations in these two genes spontaneously induced HNSCC development. We have established a genetically engineered mouse model, by deleting *p53* and constitutively activating *PIK3CA* in mouse keratin 15-expressing (K15^+^) stem cells, which leads to the development of multi-lineage tumors including SCCs, termed keratin-15-p53-PIK3CA (KPPA) tumors [22]. In the current study, we established different KPPA SCC tumor lines and performed in-depth phenotypic characterization of them. We envision that these KPPA cell lines may provide an experimental model system to further elucidate how *TP53* deletion and *PIK3CA* hyperactivation cooperate to result in aggressive phenotypes of HNSCCs.

The tumor microenvironment (TME) of HNSCCs is composed of various subsets of tumor-infiltrating cells that can interact with tumor cells or with each other via intricate networks to promote tumor progression or mediate anti-tumor immune responses. We have extensively reviewed how different subsets of immune cells contribute to an immunosuppressive TME of HNSCCs [26]. In particular, myeloid cells such as myeloid-derived suppressor cells (MDSCs) and tumor-associated macrophages (TAMs) in the TME not only promote tumor progression and angiogenesis, but also suppress anti-tumor immune responses [26]. MDSCs are CD11b^+^ cells and can be phenotypically subdivided into two groups, polymorphonuclear MDSC (PMN-MDSC) and monocytic MDSC (M-MDSC) [27]. One of the major functions of PMN-MDSCs is to suppress T cells, while M-MDSCs tend to differentiate into TAMs at tumor sites. TAMs are classified into two subpopulations: M1-TAMs, which mediate proinflammatory and anti-tumor responses, and M2-TAMs, which are immunosuppressive and promote tumor growth [26, 28]. M2-TAMs express a higher level of CD206 and display immunosuppressive properties by expressing arginase-1 (Arg-1), chemoattractant such as IL-10 and TGF-β, and chemokine CCL17 and CCL22 [29]. Prior studies showed that HNSCC TME largely encompasses M2-TAMs, which may impair effector T cell function [30]. A higher level of TAMs in the TME correlates with lymph node metastasis and advanced stage of HNSCCs [26, 28]. While it is conceivable that tumor-derived growth factors or cytokines may be able to modulate the TME, it remains incompletely understood how SCCs harboring genetic alterations in both *TP53* and *PIK3CA* drive the expansion of TAMs.

Immune checkpoint inhibitors (ICIs), including monoclonal antibodies against programmed death 1 (PD1) and PD ligand 1 (PD-L1), have been approved for HNSCCs; however, different patients exhibit highly variable responses, and the overall response rate remains low [31–38]. In addition, reproducible and highly reliable markers are still lacking to predict ICI responses in HNSCCs. In the current study, we established two different KPPA tumor lines that mimic human HNSCCs with dual genetic alterations in *TP53* and *PIK3CA*, and found they upregulated distinct signaling pathways. Moreover, we showed that these two KPPA tumor lines responded to anti-PD-L1 differentially, although both were initiated by the same oncogenic driver mutations. Our study indicates the limitations of stratifying cancers according to their genetic alterations and suggests that evaluating HNSCC tumor-intrinsic cues along with immune profiles in the TME may help better predict ICI responses.

## Materials and methods

### Generation of tumor cell lines and in vivo mouse work

The parental TAb2 and TCh3 cell lines were derived from spontaneous tumors that developed in the same female K15.CrePR1(+)p53^f/f^PIK3CA^c/c^ mouse [22] at different locations (Fig. S1A). Then, the parental KPPA tumor lines were transplanted into wildtype (WT) C57BL/6 (B6) recipients (Jax Laboratories) (Fig. S1A). Transplanted tumors were isolated and used for histology analysis and for creating daughter TAb2 and TCh3 cell lines that were employed for all the tumor injection studies (Fig. S1A). TAb2 and TCh3 cells were cultured in DMEM complete media supplemented with 10% fetal bovine serum (FBS), 1% penicillin/streptomycin, 1% HEPES buffer at 37 °C CO_2_ incubator (5%).

Tumor cells (0.5 × 10^6^ TAb2 or 1 × 10^6^ TCh3) were injected into wild-type (WT) female C57BL/6 (B6) mice (Jackson Laboratories) (6–8 weeks old). Mice were injected subcutaneously at their flank with tumor cells suspended in PBS and 50% Matrigel Basement Membrane Matrix (Corning) to a final volume of 100 μl. When tumor volume reached about 150-200 mm^3^ approximately 9–12 days post-injection, subsequent treatment was initiated. Tumor-bearing mice were treated with anti-PD-L1 (clone 10F.9G2, BioXCell, Catalog# BE0101) by intraperitoneal (i.p.) injection of 200 μg/mouse/time diluted in PBS for 2 weeks (three times per week). PBS only was used as vehicle control (VC). Tumor length and width were measured with calipers and tumor volume was calculated as length×width^2^ × *(π*/6). Relative tumor volume (RTV) was used to assess treatment effects, defined as TVn/TV0, where TVn is the TV at day n and TV0 is the TV when the treatment started. Recipient survival was monitored until mice reached endpoints of severe tumor ulceration, tumor volume reaching 20 mm in diameter or other humane end points, and mice were euthanized in accordance with institutional guidelines. All mice were maintained under specific pathogen-free conditions in the vivarium facility of University of Colorado AMC. Animal work was approved by the Institutional Animal Care and Use Committee of University of Colorado Anschutz Medical Campus (Aurora, CO).

### In vitro culture with Bone Marrow (BM) cells and tumor cells

BM cells were collected from WT B6 mice and obtained by using a 25-G needle and syringe as described previously [39]. BM cells were filtered through 70 μm cell strainer and red blood cells (RBC) were lysed with RBC lysis buffer (Sigma Aldrich, USA). BM cells were then counted (1 × 10^6^) and co-cultured with either TAb2 or TCh3 tumor cells (2.5 × 10^4^) in 24 well plates. Tumor cells were seeded in DMEM complete media in three 24-well plates 24 h prior to BM collection (Day − 1) for analysis at different time points (Day 2, 3, and 4). The supernatant of tumor culture was removed the next day (Day 0), and BM cells (1 × 10^6^) in 1 mL RPMI complete media were added. Media only with BM cells was used as control. Co-cultured cells were collected on different time points, stained for myeloid cell populations, and analyzed by flow cytometry (BD Fortessa). For transwell co-culture, TAb2 or TCh3 tumor cells (2.5 × 10^4^) in 200 μl DMEM media were placed in the top insert of a transwell (Corning, CLS3413-48EA), while BM cells (1 × 10^6^) in 800 μl RPMI media were seeded on the bottom well. Cells cultured for different time points (Day 2 and 4) were collected and analyzed as described above.

For inhibiting CSF1R or VEGFR, 10 μg/mL of anti-CSF1R mAbs (BioXCell, BE0213) or 500 nM/mL of Axitinib (MedChemExpress, HY-10065) were added into the co-culture of TAb2 tumor and BM cells, respectively. TAb2 cells co-cultured with BM cells alone were used as control. Co-cultured cells were collected on different time points (Day 2, 3, and 4) and analyzed as described above. On Day 2, one plate was used for staining while the other plates were replenished with their corresponding conditioned medias. Subsequently, co-cultured cells were collected on Day 3 and 4 for analysis as described above.

### Immune profiling by flow cytometry

Single cell suspensions were prepared from spleens and tumors harvested from WT B6 tumor-bearing mice as previously described [22]. Single cell suspensions of tumor samples were prepared by finely cutting the tumors with surgical blades into smaller pieces. Then Liberase DL (50 μg/ml) was added to the diced tumor suspensions, and incubated at 37 °C for 30 min. Then, Liberase was neutralized with 2% FBS medium, and tumor suspensions were filtered through 70 μm cell strainers, and centrifuged at 1500 rpm for 5 min at 4 °C to obtain a pellet. Cell pellets were resuspended in culture media and are ready to be stained. Single-cell suspensions were used for immediate staining with flow cytometry antibodies, or for ex vivo stimulation followed by antibody staining as previously described [22]. Briefly, cells were stained with 1:1000 LIVE/DEAD™ Fixable Aqua Dead Cell Stain (Invitrogen). Cells were then washed twice with 2% FBS in PBS before adding TruStain FcX™ (anti-mouse CD16/32) (BioLegend). Surface staining was then performed by adding Brilliant Stain Buffer Plus (BD Horizon) into each surface antibody flow panel mixture according to manufacturer’s instructions. BD Cytofix/CytoPerm buffer kit (BD Biosciences) was used according to the manufacturer’s instructions before adding intracellular staining antibodies for each panel. Surface and intracellular staining antibodies are listed in Table 1. Data were acquired on BD Fortessa and analyzed with FlowJo™ software V10 (FLOWJO, Oregon, USA).

**Table 1:** Antibodies used in this study

**Flow Antibodies**					
**Antibody**	**Fluorophore**	**Company**	**Catalog**	**Clone**	**Concentration**
CD11c	PerCP/Cy5.5	BioLegend	117327	N418	1 μg/mL
PD-L1	BV786	BD Bioscience	741014	MIH5	1 μg/mL
MHCII	BV711	BioLegend	107643	M5/114/15/2	0.25 μg/mL
CD19	Brilliant Violet 605	BioLegend	115539	6D5	1 μg/mL
Ly6C	BV421	BioLegend	128031	HK1.4	1 μg/mL
Ly6C	FITC	BioLegend	128006	HK1.4	1 μg/mL
Ly-6G	APC/Cy7	BioLegend	127623	1A8	1 μg/mL
CD11b	Alexa Fluor 700	BioLegend	101222	M1/70	1 μg/mL
CD206	PE/Cy7	BioLegend	141719	C068C2	1 μg/mL
CD86	BV421	BioLegend	105031	GL-1	1 μg/mL
F4/80	PE Dazzle	BioLegend	123145	BM8	1 μg/mL
TCR beta	BV605	BioLegend	109241	H57–597	1 μg/mL
CD4	BV421	BioLegend	100563	RM4–5	1 μg/mL
CD8a	Alexa Fluor 700	BioLegend	100729	53–6.7	1 μg/mL
CD45	BUV395	BD Bioscience	564279	30-F11	1 μg/mL
TNFalpha	BV650	BD Bioscience	563943	mp6-xt22	1 μg/mL
IFN gamma	PE	eBioscience	12–7311-41	XMG1.2	1 μg/mL
Granzyme B	PE/Cy7	BioLegend	372213	QA16A02	1 μg/mL
**Western Blotting and Histology Antibodies**					
**Antibody**	**Application**	**Company**	**Catalog**	**Clone**	**Dilution**
STAT3	Western	Cell Signaling Technology	12640S	D3Z2G	1:1000
p-STAT3	Western	Cell Signaling Technology	9145S	D3A7	1:2000
GAPDH	Western	Cell Signaling Technology	5174S	D16H11	1:1000
IgG-HRP	Western	Cell Signaling Technology	7074		1:3000
F4/80	MSI	Cell Signaling Technology	70076	D2S9R	1:500
Keratin 5	MSI	Abcam	ab64081	SP27	1:200
Arginase-1	MSI	Cell Signaling Technology	93668	D4E3M	1:500

### Western blot, ELISA, and cytokine Array

Cells were harvested and lysed with Lysis Buffer M (Roche) supplemented with complete mini (Roche). Samples were then loaded onto a NuPAGE™ 4 to 12%, Bis-Tris, Mini Protein Gel (ThermoFisher) and transferred to nitrocellulose membrane. Membranes were stained with antibodies against STAT3, p-STAT3 or GAPDH diluted according to manufacturer’s recommendations. Membranes were washed and later stained with anti-rabbit IgG-HRP secondary antibody. Blots were imaged on an Odyssey 9120 Digital Imaging System (Li-Cor). All the antibody information is included in Table 1.

TAb2 or TCh3 tumors cells (1 × 10^6^) were seeded onto 100 mm culture dish and incubated for 48 h. Cell lysate and/or culture supernatant were then collected for ELISA or Proteome Profiler Mouse XL Cytokine Array. For ELISA, cell lysate or supernatant samples were analyzed for cytokine/chemokine levels using the Mouse HGF ELISA Kit (Raybiotech, ELM-HGF), Mouse CXCL17/VCC-1 ELISA Kit (Raybiotech, ELM-CXCL17), Mouse CXCL16 (Sigma-Aldrich, RAB0127), Mouse CXCL12/SDF-1α (Sigma-Aldrich, RAB0125, and Raybiotech, ELM-SDF1a), and Mouse CSF1 (Raybiotech, ELM-MCSF-1) according to the manufacturer’s instructions. Buffer alone served as background and was subtracted from OD reading of 450 nm.

Proteome Profiler Mouse XL Cytokine Array (R&D, ARY028) was performed according to the manufacturer’s instructions. Cytokine array membranes were imaged on an Odyssey 9120 Digital Imaging System (Li-Cor). Data was analyzed by capturing the pixel density (signal) and the signals were then normalized by subtracting the background to calculate the intensity of each cytokine. GraphPad Prism 9.1.2 software (GraphPad Software, Inc.) was used to analyze data.

### Histology analysis, immunofluorescence (IF) and multispectral imaging (MSI) staining

Hematoxylin and eosin (H&E) and immunofluorescence (IF) staining of tumor tissues were performed as described previously [22]. For analyzing the spatial immune profile of mouse tumor tissues by MSI, the Opal™ 4-Color Fluorescent IHC Kit (Akoya Biosciences, NEL810001K) was used according to the manufacturer’s instructions. Slides were stained with primary antibodies against F4/80, Keratin 5, and Arginase-1 (see Table 1).

### Survival analysis of TCGA HNSCC patient cohort

Within cBioPortal platform (https://www.cbioportal.org) and under the category of head and neck cancers, we downloaded data from two cohorts of HNSCCs (TCGA, Firehose Legacy, *n* = 530; and TCGA, PanCancer Atlas, *n* = 523 samples), including clinical data, DNA mutation data, normalized mRNA expression data (log-transformed mRNA expression z-scores compared to the expression distribution of all samples) and Copy Number Alteration (CNA) data. Data from those two cohorts were merged by utilizing patient IDs (*n* = 527) and patients who have both amplification or gain of PIK3CA copy number (PIK3CA^Amp^) and truncation (include nonsense mutation, frame shift insertion and frame shift deletion) or missense of TP53 gene (TP53^Mutated^) were filtered out for the later survival analysis (*n* = 305). However, only 300 patients had analyzable data due to 5 of them missing mRNA expression data. Scores were calculated based on sum of normalized expression for each of the genes, and PIK3CA^Amp^/TP53^Mutated^ HNSCC patients were divided into high-expression group (having a score > the median score) or low-expression group (having a score < = the median score). The association between different group survival was evaluated by cox regression and the *p*-value was presented within Kaplan-Meier curves.

### Bulk RNA-sequencing, Whole Exome Sequencing (WES), and single cell RNA-sequencing

TAb2 and TCh3 tumor cells were cultured in DMEM complete media and collected for RNA purification. Total RNA was purified with TriPure (Roche) and cleaned up with RNAeasy Kit (Qiagen) according to manufacturer’s instructions. RNA samples were then depleted of ribosomal RNA and subjected to pair-ended RNA sequencing by NovaSEQ 6000 (University of Colorado at Anschutz Genomics and Microarray Core). Raw sequencing data with adapter sequences were filtered using BBduk from BBtools (version 38.86) to remove adapter contamination and obtain clean data for subsequent processing. To obtain transcript quantification from RNA-seq data, alignment tool Salmon was employed. Output files from Salmon were used for visualization and further analysis. Output files were processed using the function DESeqDataSetFromTximport in DESeq2 (V.1.30.1) to create DESeq objects and were normalized to identify differentially expressed genes (DEGs). To remove noise values associated with low count genes, the function lfcShrink in DESeq2 using apeglm estimator was applied to shrink log_2_(fold change) [40]. Volcano plot and heatmap were created with ggplot2 and pheatmap packages in R.

Genomic DNA was purified from TAb2 or TCh3 tumor cells and DNA samples were submitted to Novogene for WES using library preparation kit (Agilent SureSelect Mouse All Exon). The library was checked with Qubit and real-time PCR for quantification and bioanalyzer for size distribution detection. Quantified libraries were pooled and sequenced on Illumina NovaseqS4 PE150. Quality control was performed using FastQC. Reads were aligned to the mouse reference genome (mm10/GRCm38) using Burrows-Wheeler Aligner (BWA) [41] and the aligned files were further sorted and marked for duplicates by Picard tools. Base quality scores were recalibrated by Genome Analysis Toolkit (GATK, version 4.2.2.0) BaseRecalibrator. Subsequently, two variant-calling pipelines were applied to identify tumor-specific variants. For the first pipeline, GATK (version 4.2.2.0) was applied as follows: Mutect2 function was used to call unique variants by comparing two cell lines. The TAb2 unique variants were called by considering TAb2 as tumor tissue and TCh3 as normal control, while TCh3 unique variants were called vice versa. For the second pipeline, BCFtools *mpileup* function [42] was applied to call variants per site. Mutation callings of the two cell lines were merged and compared. The unique variants for TAb2 compared with TCh3 were defined as: TAb2 (tumor) total count > = 10, TAb2 alternative count > = 4, TAb2 alternative rate > =10% and TCh3 (normal) alternative rate = 0. Similarly, unique variants for TCh3 were defined vice versa. Next, unique variants per cell line were annotated for SNPs and amino acid (protein) changes by tool SnpEff and SnpSift [43]. For the first pipeline, further filtering was performed with FilterMutectCalls function after annotation and only passed variants were included. Variants with high or moderate putative impact were used for further analysis that might change protein functions or effectiveness.

Single cells suspensions were obtained from tumor-bearing mice treated with PBS or anti-PD-L1 as described previously [22]. CD45^+^ immune cells were isolated using EasySep™ Mouse CD45 Positive Selection Kit (StemCell Technologies, Catalog# 18945) according to manufacturer’s instructions. Purified CD45^+^ immune cell samples were submitted to the University of Colorado at Anschutz Genomics and Microarray Core for single cell capture and library preparation. Cells were loaded into a 10 × Genomics Single-cell Chip G for the 3′ captures. Single-cell gene expression libraries were prepared using Chromium Next GEM Single Cell 3′ Reagent Kits (v3.1: Dual Index Libraries) according to the manufacturer’s instructions. Samples were sequenced on the Illumina NovaSeq 6000 platform for an estimated read depth of 100,000 reads per cell. After sequencing, reads were mapped to the reference mm10 genome using the 10 × Genomics CellRanger (V.2.0.2, V.3.0.2 and V.3.1.0) count pipeline. Cells with < 500 genes detected or > 10% mitochondrial RNA content were removed from further analysis. Samples were processed using the functions NormalizeData, FindVariableGenes and ScaleData in Seurat V3.2.3. Integrated variable features were used to cluster and visualize all cells by UMAP with RunUMAP, FindNeighbors, and FindClusters in Seurat V3.2.3. Each cluster was defined by comparing their gene expression to single-cell RNA sequencing databases of known cell types [44] and PanglaoDB [45] along with a curated list of commonly known markers. Clusters were then renamed and visualized by their UMAP coordinates. UMAPs, bar graphs and violin plots were created with the R packages. DEGs between anti-PD-L1 treated and control were identified by FindMarkers function in Seurat V4.0.3 and used to find enriched pathways by using Gene Ontology (GO) analysis in clusterProfiler (V.4.0.2).

### Ingenuity Pathways Analysis (IPA)

Pathway analysis was performed with the QIAGEN’s Ingenuity® Pathway Analysis (IPA®, QIAGEN Redwood City, www.qiagen.com/ingenuity) software. Canonical pathways significantly enriched among the DEGs in the dataset were identified using one-sided Fisher’s Exact Test and the Benjamini-Hochberg method was used to adjust canonical pathway to obtain FDR *p*-values (significant threshold at 0.05).

## Results

### Different KPPA tumors responded to anti-PD-L1 treatment differentially

Different parental KPPA tumors were generated from K15.CrePR1(+)p53^f/f^PIK3CA^c/c^ mice as described previously [22]. The parental TAb2 and TCh3 tumors originated at different locations from the same female K15.CrePR1(+)p53^f/f^PIK3CA^c/c^ mouse, which were used to derive parental TAb2 and TCh3 tumors cell lines (Fig. S1A). We then transplanted these parental tumor cell lines into wildtype (WT) C57BL/6 (B6) mice and isolated the transplanted tumors for subsequent analysis and for establishing daughter TAb2 and TCh3 cell lines that were employed in the current study (Fig. S1A). We also confirmed that these daughter KPPA tumor cell lines lacked TP53 protein and harbored the constitutively active *PIK3CA* allele (Fig. S1B).

**Fig. 1:**
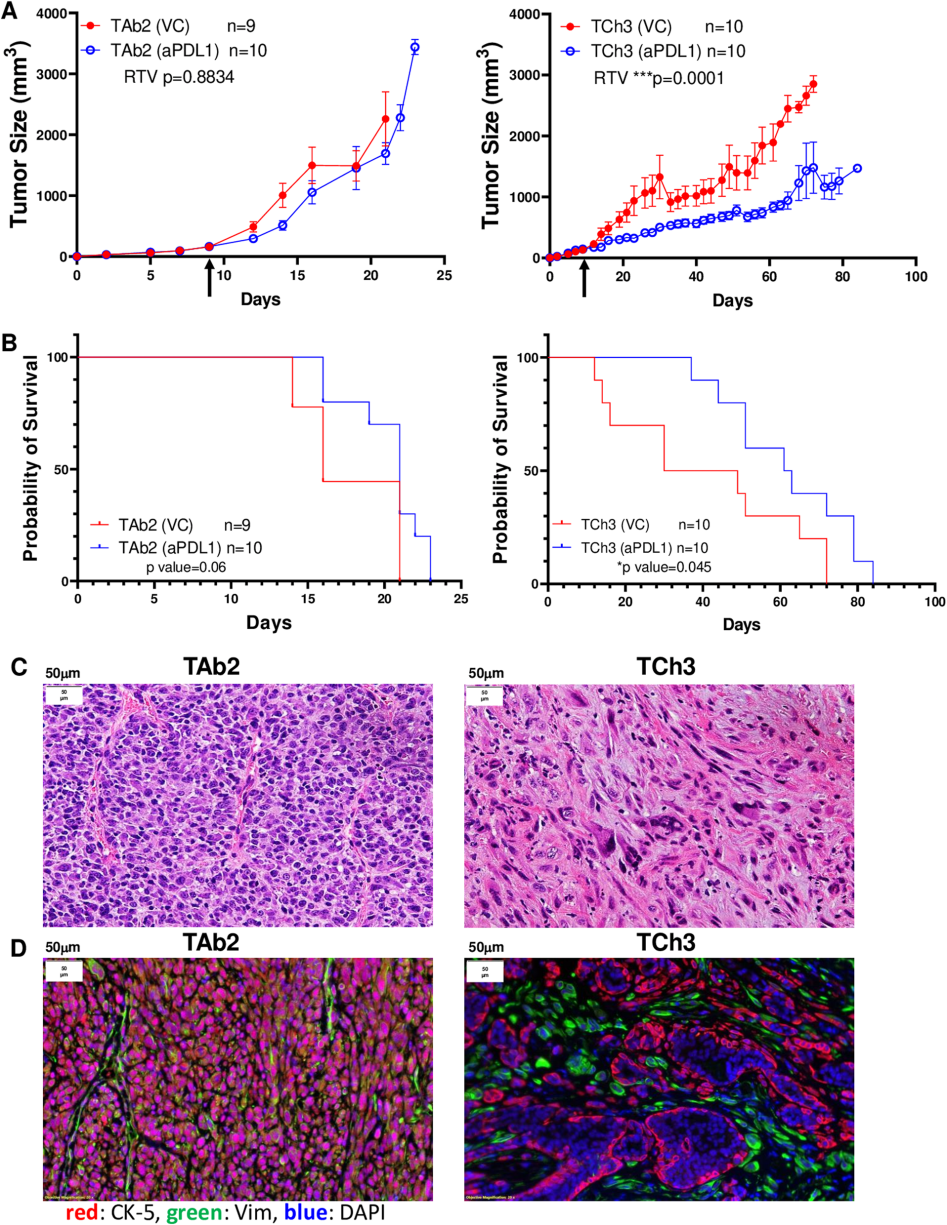
Phenotypical characterization of TAb2 and TCh3 tumors. **A** Tumor growth curves in WT B6 mouse recipients. Left panel: TAb2 vehicle control (VC) (*n* = 9) and TAb2 anti-PD-L1 treated (*n* = 10). Right panel: TCh3 VC (*n* = 10) and TCh3 anti-PD-L1 treated (*n* = 10). Treatment started when tumor size reached about 150-200 mm^3^ (indicated by arrow). Relative tumor volume (RTV defined in Method) was compared between control and treated group. Statistical significance was calculated using paired *t* test. **B** Survival curves of WT B6 mice bearing TAb2 (left) or TCh3 (right) tumors that were treated with VC or anti-PD-L1. Survival curve was compared between control and treated group using Gehan-Breslow-Wilcoxon test. **C** H&E analysis of KPPA tumor morphology. Representative images of H&E staining for daughter KPPA SCCs obtained from tumor-bearing mice, TAb2 (left) and TCh3 (right). **D** Immunofluorescence (IF) staining of KPPA tumors. Representative images of IF staining for daughter KPPA SCCs, TAb2 (left) and TCh3 (right). Cytokeratin 5^+^ (CK-5^+^) (red) tumor cells were separated from vimentin (Vim^+^) (green) stroma cells, and DAPI (blue) staining indicated nuclei (20× magnification)

To investigate the effects of immunotherapy on these tumor models in vivo, we implanted TAb2 or TCh3 tumor cells into WT B6 mice. When tumor volume reached ∼150mm^3^, tumor-bearing mice were randomized into two groups that were either treated with PBS (vehicle control) or anti-PD-L1. We found that anti-PD-L1 treatment had no effects on TAb2 tumor growth, whereas it significantly hindered TCh3 tumor progression (Fig. 1A). Furthermore, anti-PD-L1 treatment failed to affect the overall survival of TAb2 tumor-bearing mice; however, it significantly prolonged the survival of TCh3 tumor-bearing mice (Fig. 1B).

We noticed that when mice were injected with the same numbers of tumor cells, TAb2 tumors grew much faster and more aggressively than TCh3 tumors (Fig. S1C), which highlights different growth phenotypes between these two cell lines in vivo. To further delineate the underlying mechanisms, we examined tumor morphology by H&E staining and performed immunofluorescence staining (IF) to analyze the expression of cytokeratin 5 (CK5), a marker for SCCs, and vimentin (Vim), a mesenchymal marker, both of which are important for the epithelial–mesenchymal transition (EMT) process. H&E staining data showed that TAb2 tumors were poorly differentiated (Fig. 1C), which was further validated by IF results showing TAb2 tumor undergoing EMT (Fig. 1D). Taken together, these results indicate that while TP53/PIK3CA oncogenic mutations may play a role in tumorigenesis and to an extent tumor progression, ultimately other factors (e.g., immune profiles) may play a major role in regulating ICI responses.

### Distinct immune profiles pre-existing in different KPPA tumors before anti-PD-L1 treatment

**Fig. 2:**
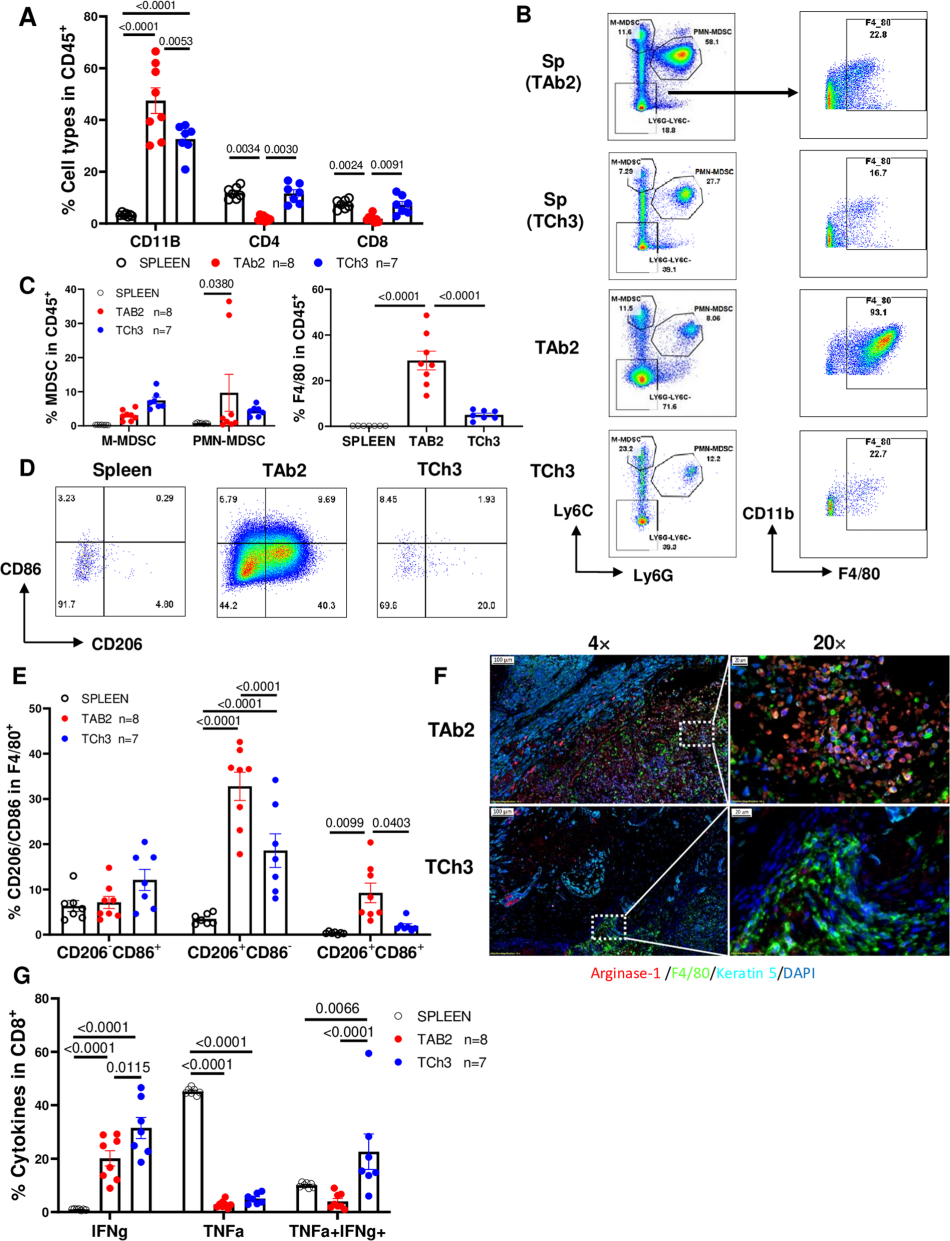
Characterization of the TME of TAb2 and TCh3 tumors. Flow cytometry analysis was performed for spleen controls (*n* = 7), or tumor-infiltrating immune cells from TAb2 (*n* = 8) and TCh3 (*n* = 7) tumors for panel **A-E** and **G**. Spleens and tumors were harvested on day 20 post-injection. Spleens from TCh3 tumor-bearing mice were used as spleen controls. **A** Quantification of the percentage of CD11b^+^, CD4^+^, or CD8^+^ cells in CD45^+^ population of spleen controls, TAb2 and TCh3 tumors. Significance was calculated using Kruskal-Wallis test. **B** Representative flow plots of different MDSC populations (left panel) and macrophages (right panel). M-MDSCs are CD11b^+^Ly6C^high^Ly6G^−^, PMN-MDSCs are CD11b^+^Ly6C^low^Ly6G^+^, and TAMs are CD11b^+^Ly6C^−^Ly6G^−^F4/80^+^. **C** Quantification of the percentages of MDSCs (left) and TAMs (right) in spleen, TAb2 and TCh3 tumors. *P* values are shown for Tukey’s multiple comparisons by two-way ANOVA (MDSCs) and one-way ANOVA (TAMs). **D** Representative flow plots for TAMs expressing CD86 and/or CD206. **E** Quantification of the percentages of M1 (CD86^+^CD206^−^) and M2 (CD86^−^CD206^+^) TAMs. *P* values are shown for Tukey’s multiple comparisons by two-way ANOVA. **F** Representative images of multispectral imaging (MSI) analysis of TAb2 (top) or TCh3 (bottom) tumors stained for Arginase 1 (red), F4/80 (green), Keratin 5 (cyan), and DAPI (blue). The white bars on the top left corners of the image indicate scale, 100 μm (left panel) and 20 μm (right panel). **G** Effector functions of CD8 TILs. The frequency of the CD8^+^ T cells producing single or double cytokines (IFNγ^+^, TNFα^+^, and IFNγ^+^TNFα^+^) in response to ex vivo stimulation. Significance was calculated using two-way ANOVA with Tukey’s multiple comparison test

We hypothesized that the pre-existing immune profiles in different tumors may underlie their differential responses to anti-PD-L1 treatment. To test our hypothesis, we examined the immune profiles in the TME of different KPPA tumors at baseline level (without anti-PD-L1 treatment). We found these two tumor cell lines exhibited distinct immune profiles in the TME. The gating strategies for different immune populations were shown in Fig. S2. TAb2 tumors contained significantly more CD11b^+^ myeloid cells than TCh3 tumors (Fig. 2A, 47.45 ± 4.95 for TAb2 vs. 32.58 ± 2.22 for TCh3). In contrast, TCh3 tumors harbored significantly higher percentages of T cells including both CD4 and CD8 T cells (Fig. 2A).

There were no significant differences in the populations of M-MDSC (Ly6C^hi^Ly6G^−^, adjp = 0.43) and PMN-MDSC (Ly6C^lo^Ly6G^+^, adj. *p* = 0.28) between TAb2 and TCh3 groups (Fig. 2B, C), likely due to the heterogenous phenotypes in TAb2 tumors, although the percentage of PMN-MDSC was higher in TAb2 tumors (9.67 ± 5.43) than spleen controls (0.76 ± 0.05) (Fig. 2C). Importantly, we found that the percentage of F4/80^+^ TAMs in Ly6C^−^Ly6G^−^ population was remarkably increased in TAb2 group compared with TCh3 group (Fig. 2B), which was also increased in CD45^+^ population (Fig. 2C, 28.82 ± 4.08 for TAb2 vs. 5.01 ± 0.76 for TCh3). In addition, the percentage of F4/80^+^CD206^+^CD86^−^ population, which represents the immunosuppressive population of M2 TAMs, was significantly higher in TAb2 tumors than in TCh3 tumors (Fig. 2D, E 32.8 ± 3.11 for TAb2 vs. 18.6 ± 3.71 for TCh3). Besides examining different populations via flow cytometry, we performed multispectral imaging (MSI) staining to evaluate the potential functions of these M2 TAMs. Our data showed that M2 TAMs produced Arginase-1 (Arg-1) in TAb2 tumors but not in TCh3 tumors (Fig. 2F), suggesting that TAb2 tumors contained not only more immunosuppressive TAMs but also more M2 TAMs with potent effector functions.

Given that TAb2 tumors exhibited such an immunosuppressive TME, we tested whether CD8 TIL’s effector functions would differ between TAb2 and TCh3 tumors. Single cell suspension was harvested from TAb2 or TCh3 tumors and cells were stimulated with PMA/Ionomycin and analyzed by flow cytometry. We found that the percentages of IFNγ^+^ and TNFα^+^IFNγ^+^ populations were significantly reduced in CD8 TILs of TAb2 tumors compared with those in TCh3 tumors (Fig. 2G). These data demonstrate that the effector functions of CD8 TILs are impaired in TAb2 tumors compared with TCh3 tumors.

### Transcriptional and genetic differences between TAb2 and TCh3 tumors

**Fig. 3:**
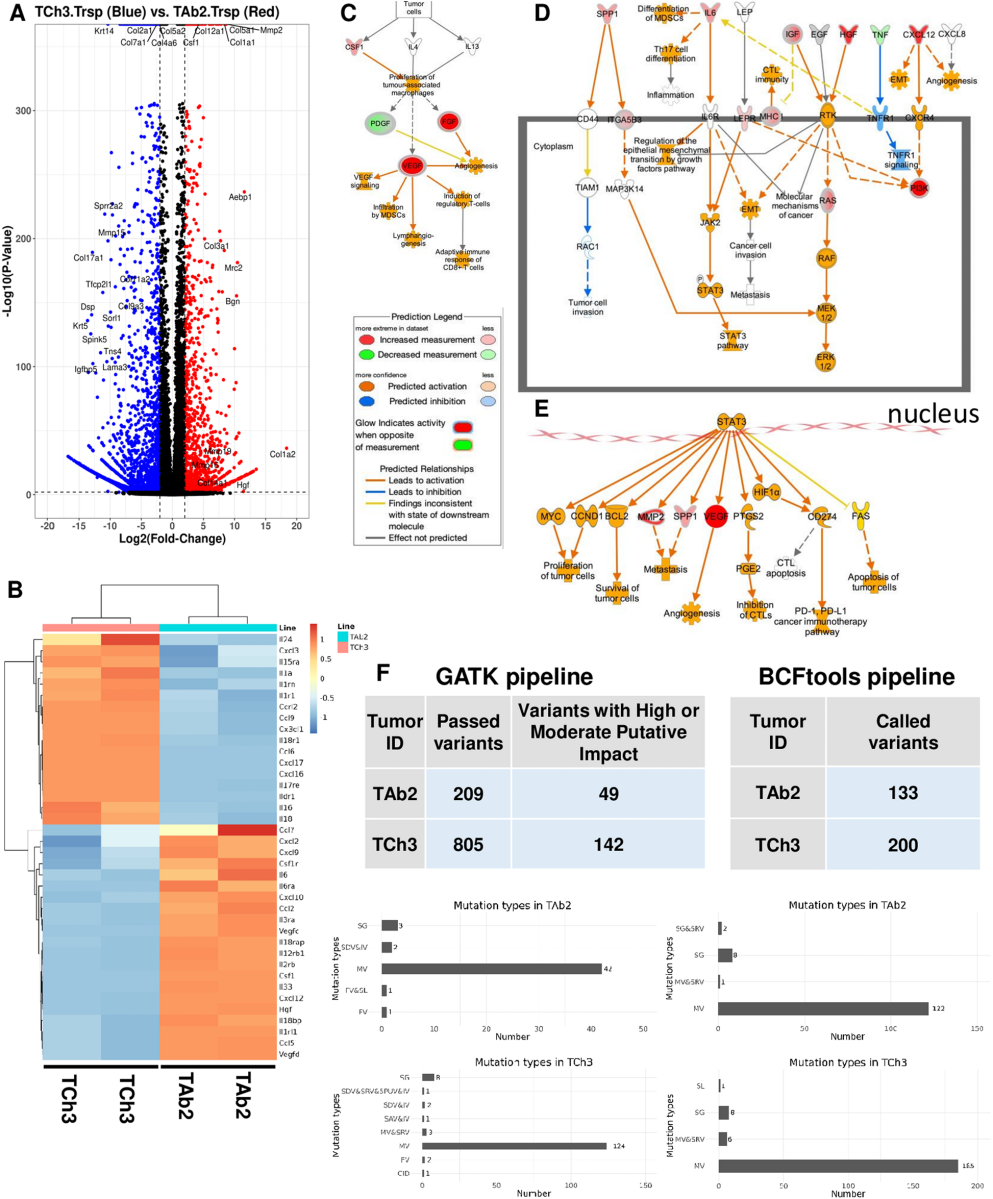
Bulk RNA-seq data of TAb2 and TCh3 tumors that differ significantly in their gene transcription and WES data of TAb2 and TCh3 tumors. **A** Volcano plot of differentially expressed genes (DEG) between TCh3 (blue) and TAb2 (red) tumors. Difference between DEGs in these two groups was plotted against a threshold of log2(fold change) = 2 and Benjamini-Hochberg (BH) adjusted *p*-value = 0.05. **B** Heatmap of gene expression of selected cytokines and chemokines. Expression values for each gene are scaled across TAb2 (*n* = 2) and TCh3 (*n* = 2) tumor cells. Genes were filtered for those differentially expressed with a threshold of log2(fold change) = 2 and BH adjusted *p*-value = 0.05. **C-E** Ingenuity Pathway Analysis (IPA) of the altered genes for corresponding pathways to identify the canonical pathways that were significantly enriched among the DEGs in TAb2 and TCh3 tumors. The key affected pathways, components, and cellular functions of the TME are presented in **(C)** tumor-secreted factors, **(D)** cytoplasm, and **(E)** nucleus of TAb2 tumors compared to TCh3 tumors. Prediction legend indicates the measurement in dataset, predicted activation or inhibition, and predicted relationships. **F** WES data showing TAb2 and TCh3 tumors exhibited genetic differences. Total numbers of tumor-specific somatic mutations in TAb2 or TCh3 tumors analyzed by two pipelines GATK (left) and BCFtools (right). Mutation type abbreviations: MV: Missense Variant; SDV: Splice Donor Variant; IV: Intron Variant; SG: Stop Gained; FV: Frameshift Variant; SL: Start Lost; SRV: Splice Region Variant; SAV: Splice Acceptor Variant; 5PUV: 5 Prime UTR Variant; CID: Conservative Inframe Deletion

To further our understanding of the unresponsiveness of TAb2 tumors to anti-PD-L1 treatment, we performed transcriptional analysis by bulk RNA sequencing (RNA-Seq) using tumor RNA samples to identify potential tumor-intrinsic factors that may contribute to an immunosuppressive TME of TAb2 tumors. A volcano plot revealed many differentially expressed genes (DEG) between TAb2 vs. TCh3 tumor cells, such as colony stimulating factor 1 (CSF1) and MMP2 upregulated in TAb2 tumors (Fig. 3A), suggesting that the enrichment of specific genes may explain the heterogeneous TME and differential ICI responses between TAb2 and TCh3 tumors. All the top DEGs between TAb2 and TCh3 that have *P* value equal to 0 were included in Table S1 (e.g., MMP2, Krt14). In addition, we observed many differentially expressed chemokines/cytokines and their receptors between TAb2 and TCh3 tumors as shown in a heatmap (Fig. 3B). We also analyzed the RNA-seq data to identify potential epigenetic modulators differentially expressed between TAb2 and TCh3 tumor cells. While there were 16 histone modifiers showing 1.5-fold difference in expression level (Fig. S3A), we did not observe many epigenetic modulators showing 2-fold difference in expression between TAb2 and TCh3 tumors (Fig. S3B). Altogether, these data suggest that tumor-intrinsic factors from responder cell line (TCh3) and non-responder cell line (TAb2) may help distinguish the efficacy of ICI therapy.

To delineate the potential relationship of DEGs and alterations to TME signaling pathways, we performed Ingenuity Pathway Analysis (IPA) to compare the genes involved in the TME that showed significant signaling amplification or reduction. IPA identified predicted pathways and networks for DEGs by TAb2 and TCh3 tumor cell lines (Fig. 3C-E). These overlapping IPA networks included vascular endothelial growth factor (VEGF)-, hepatocyte growth factor (HGF)- and Signal transducer and activator of transcription 3 (STAT3)-related signaling pathways (Fig. 3C-E). In comparison to TCh3, TAb2 tumor lines overexpressed the components in differentiation of monocytes and EMT/tumor progression pathways (Fig. 3C, D), for example, TAb2 tumor cells expressed an increased level of CSF1 (Fig. 3C), HGF, and CXC chemokine ligand 12 (CXCL12) (Fig. 3D) which activates downstream pathways correlated with metastasis and survival/proliferation of tumor cells [46–49]. VEGF pathway was predicted to be activated in TAb2 tumors (Fig. 3C, E), and VEGF has been shown to promote monocyte recruitment and tumor angiogenesis [50]. The STAT3 pathway was also predicted to be activated in TAb2 tumors (Fig. 3D), which has been shown to inhibit the differentiation of monocytes into DCs [51] and further activate VEGF [52, 53] and MMP2 [54] (Fig. 3E). Overall, these findings underscore potentially important differences in transcriptomic changes in the components of specific signaling pathways, which may enable TAb2 tumors to establish a more immunosuppressive TME and become unresponsive to anti-PD-L1 therapy.

Furthermore, we performed whole exome sequencing (WES) of TAb2 and TCh3 tumor lines and identified genetic differences between these two tumor lines in the WES data that were independently analyzed using two different pipelines (see details in Method). Both analyses showed that TAb2 tumors contained tumor-specific somatic mutations while TCh3 tumors harbored even more of such mutations (Fig. 3F). We focused on nonsynonymous mutations that cause protein changes and found that most of them were missense variants (Fig. 3F). All of the identified somatic mutations were listed in Table S2 and S3 (analysis I, GATK pipeline) and Table S4 and S5 (analysis II, BCFtools pipeline). We also identified overlapping mutations detected by both pipelines (Fig. S4, Table S6 and S7). Hence, we conclude that TAb2 and TCh3 tumors harbor tumor-specific genetic differences that may underlie their differential phenotypes.

### Drastically increased TAMs dependent on tumor-derived CSF1 and VEGF

**Fig. 4:**
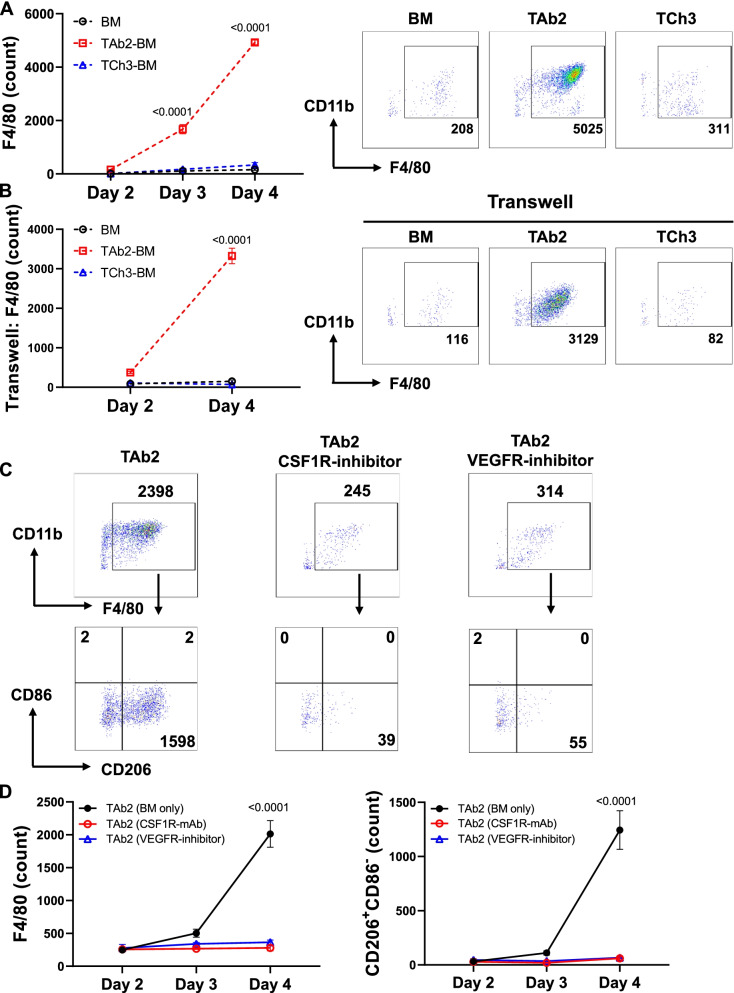
TAb2 tumors promote drastic expansion of F4/80^+^ TAMs. An in vitro co-culture assay was set up using BM cells and TAb2 or TCh3 tumor cells, for evaluating the effects of tumors on myeloid cells. Total numbers of TAMs (CD11b^+^Ly6C^−^Ly6G^−^F4/80^+^) were counted by flow cytometry at different time points (day 2, 3 and 4). **A** TAb2 tumors drive the expansion of TAMs. BM cells were either cultured alone (BM) or co-cultured with TAb2 (TAb2-BM) or TCh3 (TCh3-BM) tumor cells, respectively. Left panel: Growth curves of F4/80^+^ TAMs. Right panel: Representative flow plots of CD11b^+^F4/80^+^ population. **B** TAb2 tumors drive the expansion of TAMs independent of cell-cell contact. BM cells were either cultured alone or cultured with TAb2 or TCh3 tumor cells, respectively, in transwell plates. Left panel: Growth curve of F4/80^+^ TAMs. Right panel: Representative flow plots of CD11b^+^F4/80^+^ population. *P* values are shown for multiple comparisons to TAb2-BM by two-way ANOVA in (**A**) and (**B**). **C-D** TAb2 tumor-mediated TAM expansion requires CSF1 and VEGF. **C** Representative flow plots of CD11b vs F4/80 (top) and CD86 vs. CD206 (bottom) in the co-culture of TAb2 tumor cells and BM cells in the absence or presence of CSF1R mAb or VEGFR inhibitor. **D** Growth curves of F4/80^+^ TAMs (left) and CD206^+^CD86^−^ M2 TAMs (right). BM cells were co-cultured with TAb2 tumor cells in the absence (black) or presence of CSF1R mAb (red) or VEGFR inhibitor (blue). Results are representative of more than three independent experiments done in triplicates. Statistical significance was calculated using two-way ANOVA with Tukey’s multiple comparison test

To further elucidate the underlying mechanisms for the increased TAMs in TAb2 tumors, we established a co-culture system by employing bone-marrow (BM) cells that would contain myeloid precursors cultured in the absence or presence of TAb2 or TCh3 tumor cells. Our results showed that the number and percentage of CD11b^+^F4/80^+^ TAMs were markedly increased when BM cells were cultured with TAb2 tumor cells in a time-dependent manner (Fig. 4A, Fig. S5A). In contrast, BM cells alone or BM cells co-cultured with TCh3 tumor cells did not result in the increase of F4/80^+^ TAMs (Fig. 4A, Fig. S5A). We observed a variable percentage of different populations of myeloid cells (e.g., M-MDSC) upon co-culture with TAb2 or TCh3 tumor cells (Fig. S5B-E), albeit lacking a consistent pattern.

Next, we investigated whether the increase of F4/80^+^ TAMs population derived from cell-cell interaction between tumors and BM precursors or from tumor-derived secretory factors. To address this question, we physically separated tumor cells seeded in the top insert of a transwell and BM cells that were plated in the lower chamber in media only condition. We found that co-culturing BM precursors with TAb2 tumor cells still led to a significant increase of CD11b^+^F4/80^+^ TAMs in the transwell system, whereas BM only or BM cells co-cultured with TCh3 tumor cells failed to do so (Fig. 4B). These data suggest that TAb2 tumor-derived secretory factors probably play a major role in inducing a significant increase of TAMs.

To further dissect the tumor-intrinsic cue that may contribute to the immunosuppressive TME of KPPA tumors, we performed RNA-seq and cytokine/chemokine array. Based on these data, we chose to focus on CSF1 (a.k.a. M-CSF) and VEGF, both of which were found to be highly expressed in TAb2 tumors (Fig. 3B) and implicated in macrophage proliferation, differentiation, and recruitment [28, 55, 56]. To test whether CSF1 or VEGF plays a role in promoting TAMs in our SCC models, we employed the same co-culture system and inhibited CSF1/CSF1R-pathway or VEGF-pathway. We either introduced CSF1R inhibitor (a monoclonal antibody (mAb) against CSF1R, anti-CSF1R) or VEGFR inhibitor (Axitinib, a multi-targeted tyrosine kinase inhibitor for VEGFR) into the co-culture system. We found that inhibiting CSF1R or VEGFR significantly reduced the number of CD11b^+^F4/80^+^ TAMs and particularly the number and percentage of M2 TAMs (F4/80^+^CD206^+^CD86^−^) (Fig. 4C, D) generated from co-culture with TAb2 tumor cells. Taken together, our results suggest that increased TAMs, and specifically M2 population, were attributed to TAb2 tumor-derived CSF1 and VEGF. These findings also corroborate our in vivo data showing that TAb2 tumors contained a higher percentage of M2 TAMs.

### Validation of RNA-seq study revealed distinct pattern of chemokine/cytokine/growth factor expression in TAb2 vs. TCh3 tumors

**Fig. 5:**
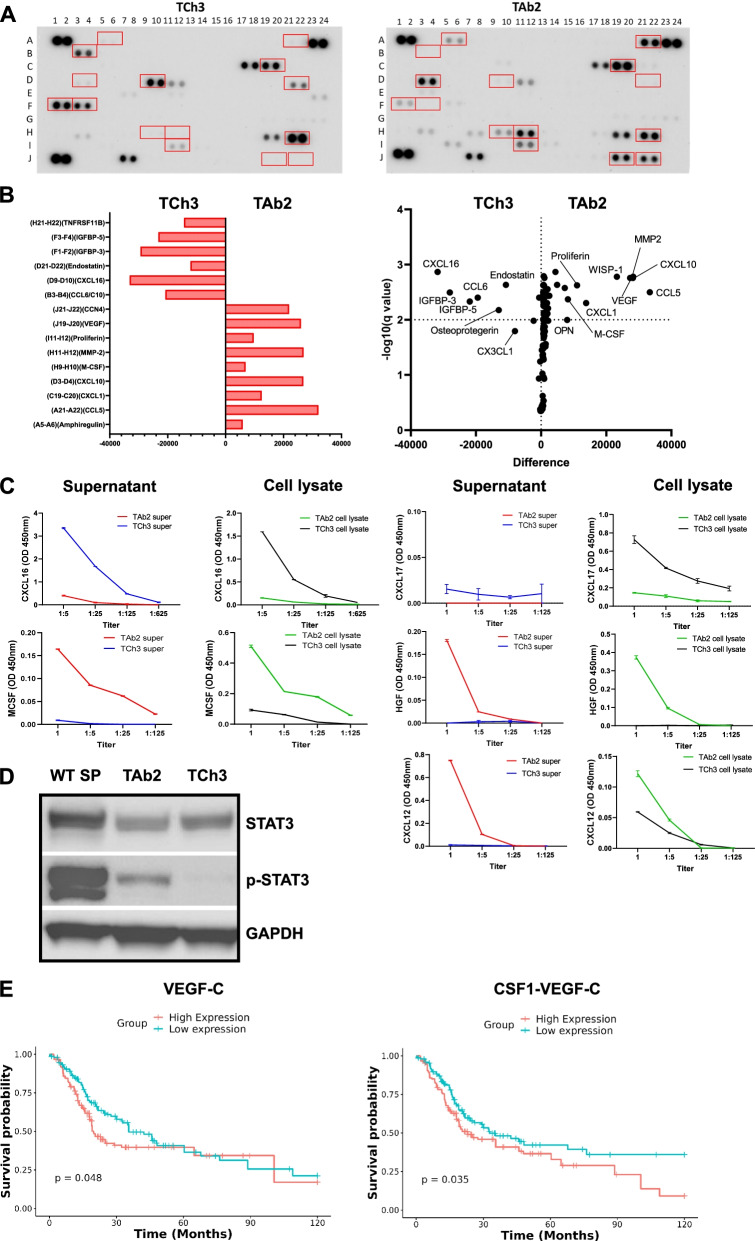
Validation assays show that TAb2 and TCh3 tumors upregulated distinct factors. **A** Representative images of cytokine array analysis of supernatants of TCh3 (left) and TAb2 (right). Red boxes indicate the cytokines that were visually different between TCh3 and TAb2 tumors. **B** Quantification of signal differences in cytokine array analysis indicated by the red boxes in (**A**). Left panel: bar graph showing the signal differences between TCh3 and TAb2 tumors. Cytokines shown vertically on the y-axis and signal intensity shown horizontally on the *x*-axis (positive values for upregulation in TAb2 and negative values for upregulation in TCh3, respectively). Right panel: volcano plot showing adj. *p*-value of the differences on the y-axis and signal intensity shown horizontally on the *x*-axis. **C** Validation of cytokine expression by ELISA. Expression of CXCL16, CXCL17, CSF1 (a.k.a. MCSF), HGF, and CXCL12 from TAb2 or TCh3 cell lysate or supernatant were detected by ELISA. The optical density (OD) at 450 nm is shown on the y-axis and the dilution factor (5-fold serial dilution) is shown on the x-axis. Results are representative of experiments done in duplicates. **D** TAb2 tumors increased the expression of phosphorylated STAT3 (p-STAT3). Western blotting analysis of total STAT3 and p-STAT3 (Tyr705) in TAb2 and TCh3 cell lysates. GAPDH was used as loading control. Data are representative of at least three independent experiments. **E** Kaplan-Meier plots of 10-year survival in PIK3CA^Amp^TP53^Mutated^ HNSCC patients (*n* = 300) expressing different levels of VEGF-C or both CSF1 and VEGF-C. Patients were grouped into high-expression group or low-expression group based on gene expression as described in Methods

To validate our RNA-seq data, we performed chemokine/cytokine protein array analysis. Our data showed that TAb2 and TCh3 tumors upregulated different sets of chemokines and growth factors (Fig. 5A, B). For instance, TAb2 tumor cells expressed a much higher level of VEGF, MMP2, CSF1 (a.k.a. M-CSF), CCN4 (a.k.a. WISP-1), CXCL10 and CCL5, consistent with RNA-seq data and IPA prediction described above. In contrast, TCh3 tumors upregulated TNFRSF11B (a.k.a. osteoprotegerin or OPG), IGFBP-3, IGFBP-5, CXCL16, CCL6, CX3CL1 and Endostatin (Fig. 5B).

Besides protein array, we validated our findings with another independent method, ELISA, using tumor cell lysate or supernatant. Our data showed that both culture supernatant and cell lysate of TCh3 tumor cells contained a higher level of CXCL17 and CXCL16 than those of TAb2 tumor cells (Fig. 5C), in line with our RNA-seq data (Fig. 3B). In contrast, both culture supernatant and cell lysates of TAb2 tumor cells expressed a higher level of CSF1 (a.k.a. M-CSF) and HGF (Fig. 5C), consistent with our RNA-seq analysis (Fig. 3B). HGF upregulation has also been shown to activate focal adhesion pathway [57, 58], consistently, our gene set enrichment analysis (GSEA) using Kyoto Encyclopedia of Genes and Genomes (KEGG) pathway showed that genes expressed in TAb2 tumors were enriched in the focal adhesion pathway (Fig. S6A). Gene concept network depicted the genes involved in the enriched pathways in TAb2 tumors (Fig. S6B). GSEA also showed that TAb2 tumors expressed genes enriched in protein processing in endoplasmic reticulum, ribosome, and PI3K-Akt signaling pathway (Fig. S6C-H). Lastly, we also verified the increased expression of CXCL12 by ELISA in TAb2 compared to TCh3 tumors (Fig. 5C).

Given the increased expression of VEGF and MMP2 and the predicted STAT3 activation by IPA analysis, we hypothesized that TAb2 tumors would exhibit a higher level of STAT3 activation. To test this, we examined the phosphorylation level of STAT3 (p-STAT3) using western blotting and found that TAb2 tumor indeed expressed a much higher level of p-STAT3 (Fig. 5D). Collectively, our validation studies demonstrate the distinct expression pattern of chemokines/cytokines/growth factors in TAb2 vs. TCh3 tumors that may be involved in tumorigenesis or progression and underlie their differential responses to anti-PD-L1 treatment.

Next, we analyzed a large HNSCC dataset from TCGA database as we did previously [22] and focused on PIK3CA^Amp^/TP53^Mutated^ HNSCC patients. Kaplan Meier curves of 10-year survival were shown for 2 different groups, high vs. low expression of VEGF-C or both CSF1 and VEGF-C (see Method) (Fig. 5E). We found that PIK3CA^Amp^/TP53^Mutated^ HNSCC patients who expressed a higher level of VEGF-C or both CSF1 and VEGF-C exhibited worse survival (Fig. 5E). However, we did not detect any statistical difference in PIK3CA^Amp^/TP53^Mutated^ HNSCC patients who expressed high vs. low level of CSF1, VEGF-A, VEGF-B singularly or CSF1/VEGF-A or CSF1/VEGF-B combinatorially (Fig. S7). These data suggest that combined high expression of CSF1/VEGF-C may serve as a predictive marker for worse survival in PIK3CA^Amp^/TP53^Mutated^ HNSCC patients.

### TAb2 and TCh3 tumors exhibited differential abilities to upregulate PD-L1 in response to IFN-γ stimulation

**Fig. 6:**
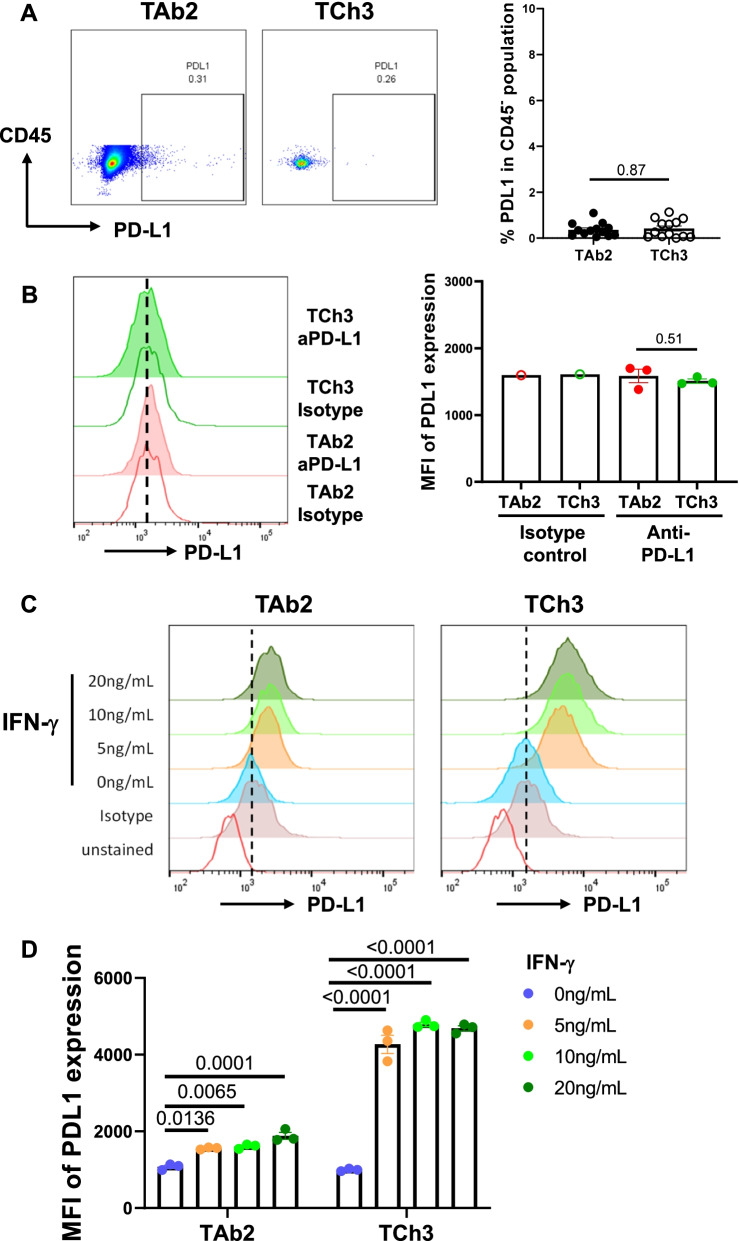
Differential ability of TAb2 and TCh3 tumors to upregulate PD-L1 in response to IFN-γ. **A** Minimal PD-L1 expression in CD45^−^ population of in vivo tumors. Representative flow plots of non-immune cells (CD45^−^ population) (left) and the percentage of PD-L1^+^ cells in CD45^−^ population of TAb2 (*n* = 13) and TCh3 (*n* = 13) tumors (right). **B** TAb2 and TCh3 tumors do not express PD-L1 at baseline. Representative histograms (left) and mean fluorescent intensities (MFI) (right) of isotype control (negative control) and anti-PD-L1 staining on in vitro cultured tumor cell lines at baseline. **C-D** TCh3 tumors exhibited enhanced responsiveness to IFN-γ. Representative histograms (**C**) and MFI of PD-L1 expression (**D**) of in vitro cultured TAb2 or TCh3 tumor cell lines either untreated or treated with different doses of IFN-γ. Representative results are shown from two independent experiments. Statistical significance is calculated from duplicates in one experiment with unpaired two-tailed *t* test. Error bars represent the SEM

PD-L1 expression has been considered as a predictive marker for ICI responses [59, 60]. To test a role of PD-L1 in our system, we examined PD-L1 expression in different types of tumor cells. First, we gated on CD45^−^ population in the TME including tumor cells and non-hematopoietic lineages for flow cytometry analysis of the in vivo tumors. We found that all of the CD45^−^ populations including tumor cells expressed a minimal level of PD-L1 and there was no significant difference in the percentage of PD-L1^+^ cells in CD45^−^ population between TAb2 (0.365 ± 0.081) and TCh3 (0.422 ± 0.109) tumors (Fig. 6A).

Because IFN-γ has been implicated in anti-tumor immunity and ICI therapeutic responses [61–63], we performed in vitro experiments by treating TAb2 or TCh3 tumor cell lines with varying doses of IFN-γ to determine their ability to upregulate PD-L1 in response to IFN-γ. We found that TAb2 and TCh3 tumor cells did not express PD-L1 at baseline (stable stage) when compared to isotype control, and there was no difference between TAb2 and TCh3 measured by mean fluorescent intensity (MFI) in their lack of PD-L1 expression (Fig. 6B). While both TAb2 and TCh3 tumor cells significantly upregulated PD-L1 expression upon IFN-γ treatment, TCh3 tumor cells drastically upregulated PD-L1 in response to IFN-γ when compared to TAb2 tumor cells (Fig. 6C, D). Hence, we conclude that TCh3 tumor cells exhibit enhanced responsiveness to IFN-γ.

### Single-cell RNA-seq delineated the changes of different immune subsets in TAb2 vs. TCh3 tumors upon anti-PD-L1 treatment

**Fig. 7:**
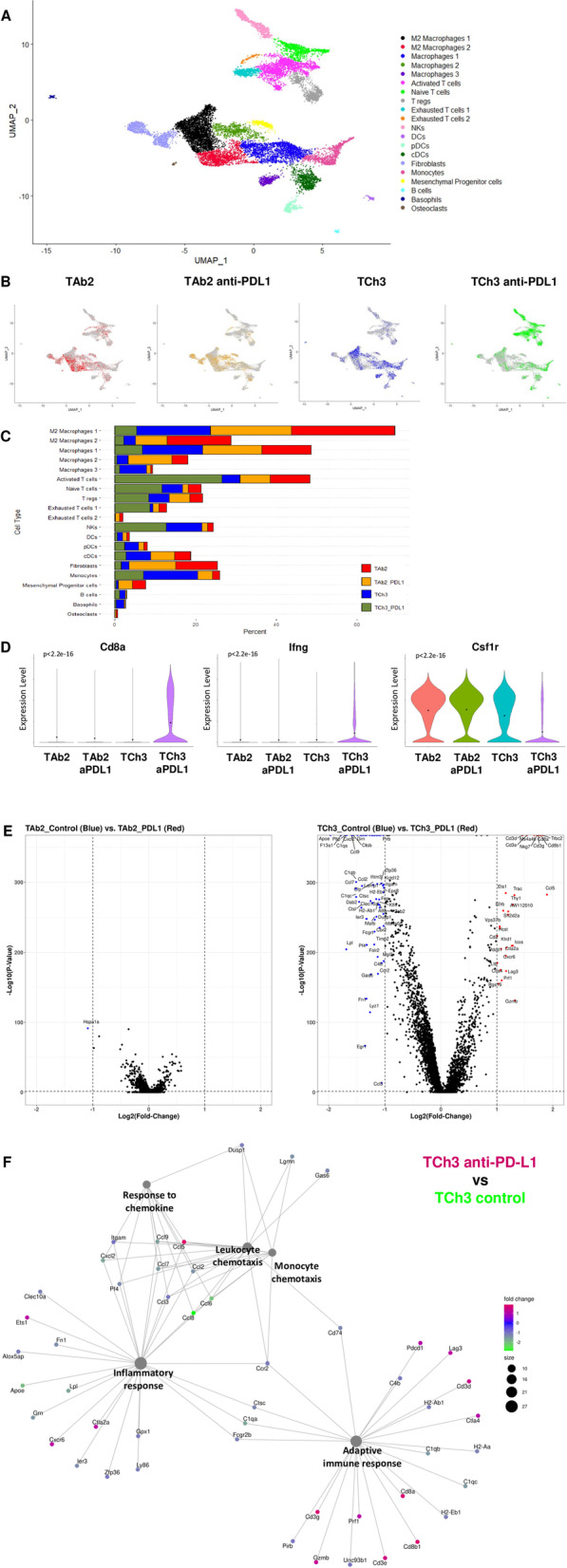
Single-cell RNA-sequencing analysis showed that anti-PD-L1 treatment increased different populations of T cells in TCh3 tumors. **A** Transcriptional data of CD45^+^ tumor-infiltrating immune cells from 4 samples (TAb2, TAb2-anti-PD-L1, TCh3, and TCh3-anti-PD-L1, *n* = 1 per sample) were integrated using Seurat’s integration algorithm and clustered using UMAP. Cluster phenotyping identified 20 functional clusters colored based on gene expression. **B** The same UMAP clusters of CD45^+^ tumor-infiltrating immune cells are shown for individual TAb2, TAb2-anti-PD-L1, TCh3 and TCh3-anti-PD-L1 sample colored based on sample type. **C** Each cluster represented as a proportion of total cells from TAb2 (red), TAb2-anti-PD-L1 (orange), TCh3 (blue) and TCh3-anti-PD-L1 (green) group. **D** Violin plots showing *Cd8a*, *Ifng*, and *Csf1r* expression in TAb2, TAb2-anti-PD-L1, TCh3 and TCh3-anti-PD-L1 group across all UMAP clusters. Gene expression level shown on the y-axis, and the binned cell count as the width shown on the *x*-axis. Black dots indicate the mean in each group. Groups were compared using one-way ANOVA. **E** Volcano plot of DEGs in CD45^+^ tumor-infiltrating immune cells between different groups. Left panel: TAb2 control (blue) vs. TAb2-anti-PD-L1 (red). Right panel: TCh3 control (blue, meaning upregulated in TCh3 control) vs. TCh3-anti-PD-L1 (red, meaning upregulated in TCh3-anti-PD-L1). Difference between DEGs in the two groups was plotted against a threshold of log2(fold change) = 1 and BH adjusted *p*-value = 0.05. **F** The category network plot shows the relationship between significant genes (threshold is defined as log2(fold change) = 1 and BH adjusted *p-*value = 0.05) and top 5 most significant GO terms in the comparison of TCh3-anti-PD-L1 vs TCh3 control group. Within each connected network, node (pathway) size is proportional to the number of neighbors (interacting genes of each node) that are upregulated in TCh3-anti-PD-L1 compared to TCh3 control. Color scheme indicates fold change of upregulation in TCh3-anti-PD-L1 (red) vs. TCh3 control (green)

To better understand the alterations in the TME of TAb2 vs. TCh3 tumors upon anti-PD-L1 treatment, we performed single-cell RNA-seq analysis using CD45^+^ immune cells isolated from tumors of untreated or anti-PD-L1 treated recipients to profile different subsets of immune cells. Single-cell RNA seq data from all samples were analyzed as described previously using Seurat v3 [64]. We clustered the CD45^+^ immune cells by uniform manifold approximation and projection (UMAP) and performed a qualitative comparison to both the SCSA and PanglaoDB datasets to verify the specific cell types (Fig. 7A). The UMAP showed two major clusters: the upper right one contained all the T cell populations and NK cells while the bottom left one mainly consisted of macrophages and monocytes (Fig. 7A). By comparing TAb2 vs. TAb2 anti-PD-L1 group, we found that anti-PD-L1 treatment had no obvious effects on different immune subsets in TAb2 tumors (Fig. 7B, C). However, anti-PD-L1 treatment drastically increased all the T cell populations including activated and naïve T cells as well as Exhausted T cells 1 population in TCh3 tumors (TCh3 anti-PD-L1) compared with all other groups (Fig. 7B, C). Furthermore, anti-PD-L1 treatment also reduced certain macrophage populations, especially M2 macrophage 1 and 2, in TCh3 tumors, when compared with other groups (Fig. 7C). In contrast, TAb2 tumors contained more M2 macrophage 1 and 2 populations regardless of anti-PD-L1 treatment, when compared with TCh3 tumors (Fig. 7C). Analysis of representative genes showed that the expression of *CD8a* and *IFN-γ* genes was significantly increased while the expression of *Csf1r* in CD45^+^ tumor-infiltrating immune cells was significantly reduced in TCh3 anti-PD-L1 group compared with other groups (Fig. 7D).

Consistent with UMAP clustering analysis, we found that anti-PD-L1 treatment did not affect gene expression in CD45^+^ tumor-infiltrating immune cells in TAb2 tumors globally shown by volcano plots (Fig. 7E, left). However, anti-PD-L1 treatment resulted in upregulation or downregulation of numerous genes in CD45^+^ tumor-infiltrating immune cells in TCh3 tumors, including *Cd3d*, *Cd3e*, *Cd3g*, *Cd8a*, *Cd8b1*, *Nkg7, Ccl5, Ets1*, *Icos*, *Cxcr6*, *Pdcd1*, *Lag3*, *Prf1*, and *Gzmb* (Fig. 7E, right). All the top DEGs between TCh3_control and TCh3_anti-PD-L1 that have *P* value equal to 0 were included in Table S8 (e.g., Cd3d, Ccl9). Gene Ontology (GO) enrichment analysis showed the top 5 pathways highly ranked in the TCh3 anti-PD-L1 group compared with TCh3 control, which include Response to chemokine, Leukocyte chemotaxis, Monocyte chemotaxis, inflammatory response, and Adaptive immune response (Fig. 7F). Taken together, our single-cell RNA-seq data suggest that the immune profiles of TCh3 tumors significantly altered and augmented different populations of T cells and corresponding gene expression upon anti-PD-L1 treatment.

### Anti-PD-L1 treatment enhanced CD8 TIL infiltration in TCh3 tumors

To validate our single cell RNA-seq findings, we examined the immune profiles in the TME of untreated or anti-PD-L1 treated tumor-bearing mice via flow cytometry. TAb2 or TCh3 tumors were implanted into WT B6 mice; when tumor size reached ~150mm^3^, tumor-bearing mice were randomized into two groups with one treated with vehicle control and another with anti-PD-L1 as described previously. Three days after the last anti-PD-L1 treatment, tumors were harvested from the recipient mice and analyzed for immune profiles. We compared the untreated control vs. anti-PD-L1 treated groups with spleen samples from tumor-bearing mice as control (SP).

**Fig. 8:**
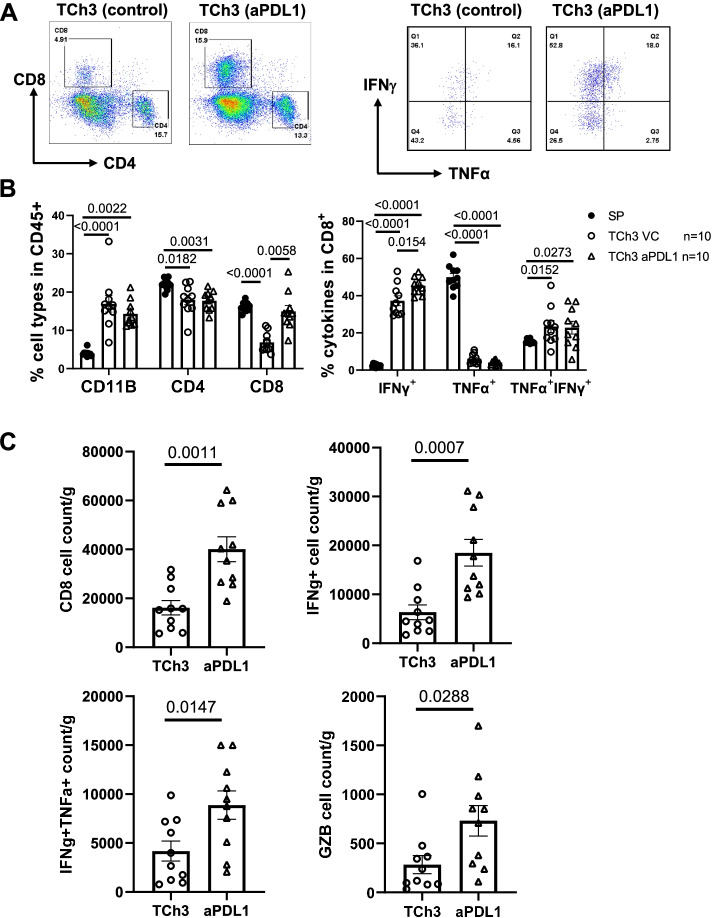
Anti-PD-L1 treatment enhanced CD8 T cell number and effector functions in TCh3 tumors. Flow cytometry analysis was performed for spleen controls (*n* = 10), or tumor-infiltrating immune cells from TCh3 VC (*n* = 10) and TCh3 anti-PD-L1 (*n* = 10) groups for all panels. TCh3 tumors were harvested on day 25 post-injection. **A** Representative flow plots of CD8/CD4 T cells (left) and CD8^+^ T cells producing cytokines (IFNγ/TNFα) (right) from TCh3 VC and TCh3 anti-PD-L1 groups. **B** Quantification of the percentage of CD11b^+^, CD4^+^, or CD8^+^ cells in CD45^+^ population (left) and frequency of the CD8^+^ T cells producing single or double cytokines (IFNγ^+^, TNFα^+^, and IFNγ^+^TNFα^+^) in response to ex vivo stimulation (right). *P* values are shown for Kruskal-Wallis test or Tukey’s multiple comparisons by two-way ANOVA. **C** Cell number count of CD8 T cells in TCh3 vs. TCh3 anti-PD-L1 group. Cell count of different CD8 populations present per gram of tumor tissue. Total CD8 (top left), IFN-γ^+^ in CD8 (top right), double IFNγ^+^/TNFα^+^ in CD8 (bottom left) and Granzyme B^+^ (GZB) in CD8 (bottom right). Statistical significance is calculated with unpaired two-tailed *t* test. Error bars represent the SEM

The immune profile of TME had no significant changes in untreated and treated TAb2 tumor-bearing mice (Fig. S8A-D). In contrast, we found that the percentage of CD8 TILs was significantly increased in TCh3 anti-PD-L1 treated group compared with control group, while the percentage of CD4 TILs did not alter between the two groups (Fig. 8A, B). Furthermore, the percentage of IFNγ^+^ single producers, but not IFNγ^+^TNFα^+^ double producers, in CD8 TILs was significantly increased in TCh3 anti-PD-L1 group compared with control group (Fig. 8A, B). The cell number counts for CD8 TIL, IFNγ^+^, IFNγ^+^TNFα^+^ and Granzyme B^+^ (GZB) populations in the TME were all increased in TCh3 anti-PD-L1 group compared with control group (Fig. 8C), consistent with our single-cell RNA seq data. Taken together, TCh3 tumors contained more CD8 TILs that also exhibited a certain degree of effector functions upon anti-PD-L1 treatment.

## Discussion

We uncovered tumor-intrinsic differences that may underlie the differential responses to ICI by establishing and employing two KPPA SCC tumor lines, TAb2 vs. TCh3, both of which harbor *TP53* deletion and *PIK3CA* hyperactivation and originated from the same K15.CrePR1(+)p53^f/f^PIK3CA^c/c^ mouse. We found that: (1) TCh3 tumors are relatively sensitive to anti-PD-L1, while TAb2 tumors failed to respond completely; (2) Prior to anti-PD-L1 treatment, the TME of TAb2 tumors is highly immunosuppressive evidenced by heavy infiltration of TAMs, especially, M2-TAMs, whereas TCh3 tumors contained more CD8 TILs with better effector functions; (3) TAb2 tumor cells drastically expanded F4/80^+^ TAMs from BM precursors, which required CSF1 and VEGF; (4) More aggressive phenotypes of TAb2 tumors correlate with upregulation of chemokines/growth factors that may contribute to immunosuppressive TME; and (5) anti-PD-L1 did not affect the TME of TAb2 tumors but significantly increased the number of CD8 TILs in TCh3 tumors. We suggest that tumor-intrinsic differences may contribute to differential ICI responses by orchestrating TME prior to ICI treatment. Although these KPPA tumors harbor same oncogenic driver mutations, they appear to establish differential TME that is highly immunosuppressive or relatively conducive for ICI therapy. These results suggest that evaluating HNSCC tumor-intrinsic cues along with immune profiles in the TME may help better predict ICI responses. Our experimental models may provide a platform for pinpointing tumor-intrinsic differences underlying an immunosuppressive TME in HNSCCs and for testing combined immunotherapies targeting either tumor-specific or TAM-specific players to improve ICI efficacy.

TAMs associate with tumor progression by promoting evasion of immunosurveillance, angiogenesis, metastasis, and therapy resistance or inhibiting effector functions of CD8 TILs [65–67]. A meta-analysis showed that increased density of TAMs, including M2-like subtypes, correlate with poor clinicopathologic markers in HNSCC such as advanced tumor stage and nodal metastasis [68]. Consistently, we found that TAb2 tumors heavily infiltrated with TAMs exhibited aggressive phenotypes and failed to respond to anti-PD-L1 completely. Furthermore, we found that co-culturing TAb2 tumor cells with BM cells resulted in drastic expansion of F4/80^+^ TAMs and CD206^+^ M2-TAMs, which is independent of cell-cell contact, suggesting a major role of secretory factors in promoting TAM differentiation. Blocking CSF1/CSF1R and VEGF/VEGFR pathways in the co-culture of TAb2-BM remarkably suppressed TAM production. In line with these findings, TAb2 tumors upregulated CSF1 and VEGF at both mRNA and protein level. While prior studies have reported that CSF1 and VEGF can stimulate differentiation and polarization of TAMs [69–71], their role in HNSCC prognosis and therapy response is less well understood. Recently, CSF1 upregulation was shown to correlate with increased TAM infiltration and poor prognosis in oral SCC [72]. Taken together, we suggest that CSF1 and VEGF upregulation may serve as predictive markers for worse prognosis and ICI therapy resistance in HNSCCs harboring *TP53* deletion and *PIK3CA* amplification.

We verified the expression level of several proteins that are involved in promoting tumor aggressiveness (e.g., invasiveness and angiogenesis) and inducing an immunosuppressive TME. We showed that TAb2 tumor cells expressed higher levels of CSF1, VEGF, HGF and CXCL12. Consistent with our findings, previous studies showed HNSCC patients with a higher level of CXCL12 have poor prognosis [73, 74]. HGF is a pleiotropic growth factor and cytokine, whose upregulation promotes tumor cell survival, motility, and proliferation [47, 48], and its receptor, HGFR (a.k.a. c-MET), is a well-known oncogene [75]. Prior studies also revealed a positive feedback loop of HGF/c-MET/STAT3 signaling that plays an important role in tumorigenesis [76–78]. STAT3 pathway is downstream of receptor tyrosine kinase (RTK) including cytokine and growth factor receptors. Cytokines such as CSF1 can activate RTK (CSF1R) and downstream STAT3 pathway, which can promote tumor migration and invasion in colon cancers [79]. In this regard, TAb2 tumors upregulated both CSF1 and CSF1R, potentially enforcing a positive signaling cycle. VEGF is another factor involved in a positive feedback loop of STAT3 activation in tumorigenesis and angiogenesis [53, 80]. IL-6/JAK/STAT3 pathway is predicted to be activated in TAb2 tumors, which is often hyperactivated in various types of cancer that correlates with poor prognosis [81]. Again, TAb2 tumors upregulated both IL-6 and IL-6Rα transcriptionally, suggesting a positive signal loop. Lastly, we found that TAb2 tumor expressed a higher level of p-STAT3. Hence, our data illustrate a central theme of positive feedback loops reinforcing aggressive phenotypes of TAb2 tumors. In contrast, CXCL17 may be one of the factors that predict less aggressive phenotypes in HNSCCs. Supporting this notion, we found TCh3 tumor expressed a higher level of CXCL17 and HNSCC patients with higher expression of CXCL17 exhibit a better prognosis [74]. Thus, our studies may provide more information for better predicting HNSCC prognosis and establish an experimental system for testing new therapeutic targets of HNSCC by breaking the vicious positive feedback cycles.

While ICI demonstrated benefits for patients with recurrent or metastatic HNSCC, the response rate remains relatively low (< 20%) [31–38]. Thus, characterizing the tumor-intrinsic signaling pathways and immune landscape that are associated with ICI-response vs. resistance may allow us to develop better strategies to improve ICI efficacy. For instance, TCh3 tumors contained more CD8 TILs prior to anti-PD-L1 treatment, the presence of pre-existing CD8 TILs may be a predictive marker for ICI efficacy [82]. Furthermore, CD45^+^ tumor-infiltrating immune cells in TCh3 tumors clearly undergo more transcriptional changes upon anti-PD-L1 treatment that favor anti-tumor immunity; however, the underlying mechanisms for these observations remain unclear. Compared with TAb2 tumors, TCh3 tumors upregulated completely different chemokines and cytokines, such as CXCL16. Interestingly, CD45^+^ immune cells in TCh3 tumors expressed more CXCR6, the only known receptor of CXCL16, upon anti-PD-L1 treatment. CXCL16 may attract CXCR6-expressing naïve CD8 or activated CD8 and CD4 T cells, NK or NKT cells [83]. On the other hand, CXCL16 was reported to positively correlate with M2-TAM infiltration, increased angiogenesis, and worse prognosis in thyroid cancer [84]. Hence, the role of different chemokines or cytokines in orchestrating TCh3 TME remains unresolved and needs to be addressed in future studies.

To test the effects of *PIK3CA* hyperactivation and *TP53* deletion on tumor-intrinsic cues regulating TME and ICI responses, we employed tumor cell lines derived from in vivo spontaneously generated KPPA SCCs [22] with these two genetic alterations. Although TAb2 and TCh3 tumors harbor the same oncogenic driver mutations for initial tumorigenesis, they exhibited differential TME and anti-PD-L1 responses, which suggest the limitations of stratifying cancers according to genetic changes and allow us to glance at vast heterogeneity potentially caused by clonal variation in cancers. In this regard, it has been reported that HNSCCs can undergo epigenetic alterations and enhance clonal variation [85–88]. We suggest that the tumor-intrinsic differences in these two cell lines may have arisen during their passage of in vivo transplantation. In line with this idea, our RNA-seq and WES data indicated tumor-specific epigenetic and genetic differences between TAb2 and TCh3 tumors that might contribute to differential responses to anti-PD-L1 by affecting tumor immunogenicity or tumor’s responses to IFN-γ stimulation. Here, we have barely begun to reveal the outcome of such intrinsic differences, it clearly requires substantial work to better understand the fundamental mechanisms defining tumor heterogeneity. Nevertheless, our studies of both tumor cells and tumor-infiltrating immune cells may help to identify new predictive markers associated with anti-PD-L1 responses and our experimental models may facilitate the testing of combinatorial immunotherapy for HNSCCs.

## Conclusions

In the current study, we uncovered tumor-intrinsic differences between two SCC tumor lines (TAb2 and TCh3), both of which harbor *TP53* deletion and *PIK3CA* hyperactivation, yet they responded to anti-PD-L1 therapy differently. Our study demonstrates that stratifying cancers according to their genetic alterations alone is not sufficient in determining ICI efficacy. In addition, our findings suggest that evaluating HNSCC tumor-intrinsic cues along with immune profiles in the TME may help better predict ICI responses in individual hosts. Our experimental models may provide a platform for pinpointing tumor-intrinsic differences underlying an immunosuppressive TME in HNSCCs and for testing combined immunotherapies targeting either tumor-specific or TAM-specific players to improve ICI efficacy. These findings may be translatable to individual HNSCC patients with unique TME (e.g., higher expression of CSF1/VEGF-C), and determining these multi-factorial profiles will help identify patients who may benefit from ICI therapy or other personalized therapies.

## Supplementary Information

**Additional file 1.**

**Additional file 2: Figure S1.** Generation of daughter cell lines, western blotting and different tumor growth pattern of TAb2 vs. TCh3 tumors. **Figure S2.** Gating strategy for flow cytometry analysis. **Figure S3.** Differential expression of epigenetic modulators in TAb2 vs. TCh3 tumor cells. **Figure S4.** WES data of TAb2 vs TCh3 tumor cells. **Figure S5.** Growth curves of different myeloid populations upon co-culture of BM cells with TAb2 or TCh3 tumor cells. **Figure S6.** Gene Set Enrichment Analysis (GSEA) using KEGG pathway depicts transcriptional profiles of negatively or positively enriched in TAb2 and TCh3 tumor cells. **Figure S7.** Survival curves for HNSCC patients expressing different levels of CSF1 and/or VEGF. **Figure S8.** Flow cytometry analysis confirmed that anti-PD-L1 treatment did not affect the cell types present in the TME of TAb2 tumors and tumor-infiltrating CD8 T cell function compared to control TAb2 tumors. **Figure S9.** Detailed *p*-values for figures.

**Additional file 3.**

**Additional file 4.**

**Additional file 5.**

**Additional file 6.**

**Additional file 7.**

**Additional file 8.**

**Additional file 9.**

**Additional file 10.**

## Data Availability

Raw data for RNA-seq, single-cell RNA-seq and WES are available in the public open access repository (NCBI database), Sequence Read Archive (SRA) and Gene Expression Omnibus (GEO). Data and material are available upon request**.**

## Abbreviations

Arg-1: Arginase-1
BM: Bone-Marrow
B6: C57BL/6
CNA: Copy Number Alteration
CSF1: Colony Stimulating Factor 1
CSF1R: Colony Stimulating Factor 1 Receptor
CK5: Cytokeratin 5
DEGs: Differentially Expressed Genes
EMT: Epithelial–Mesenchymal Transition
FBS: Fetal Bovine Serum
GSEA: Gene Set Enrichment Analysis
GO: Gene Ontology
GZB: Granzyme B
HNCs: Head and Neck Cancers
HNSCCs: Head and Neck Squamous Cell Carcinomas
HPV: Human Papillomavirus
H&E: Hematoxylin and Eosin
HGF: Hepatocyte Growth Factor
IF: Immunofluorescence
ICIs: Immune Checkpoint Inhibitors
IPA: Ingenuity Pathway Analysis
KEGG: Kyoto Encyclopedia of Genes and Genomes
K15: Keratin 15
KPPA: Keratin-15-p53-PIK3CA
mAb: Monoclonal Antibody
MDSCs: Myeloid-Derived Suppressor Cells
M-MDSC: Monocytic MDSC
OPG: Osteoprotegerin
p-STAT3: Phosphorylated Signal Transducer and Activator of Transcription 3
PD1: Programmed Death 1
PD-L1: Programmed Death Ligand 1
PMN-MDSC: Polymorphonuclear MDSC
PI3K: Phosphoinositide 3-Kinase
RTK: Receptor Tyrosine Kinase
RBC: Red Blood Cells
RTV: Relative Tumor Volume
RNA-Seq: RNA Sequencing
STAT3: Signal Transducer and Activator of Transcription 3
TAMs: Tumor-Associated Macrophages
TME: Tumor Microenvironment
TILs: Tumor Infiltrating Lymphocytes
UMAP: Uniform Manifold Approximation and Projection
VEGF: Vascular Endothelial Growth Factor
VEGFR: Vascular Endothelial Growth Factor Receptor
VC: Vehicle Control
Vim: Vimentin
WT: Wild-Type

## References

[1] Du E, Mazul AL, Farquhar D, Brennan P, Anantharaman D, Abedi-Ardekani B, Weissler MC, Hayes DN, Olshan AF, Zevallos JP (2019). Long-term survival in head and neck Cancer: impact of site, stage, smoking, and human papillomavirus status. Laryngoscope.

[2] Cramer JD, Burtness B, Le QT, Ferris RL (2019). The changing therapeutic landscape of head and neck cancer. Nat Rev Clin Oncol.

[3] Johnson DE, Burtness B, Leemans CR, Lui VWY, Bauman JE, Grandis JR (2020). Head and neck squamous cell carcinoma. Nat Rev Dis Prim.

[4] Cancer Genome Atlas Network: comprehensive genomic characterization of head and neck squamous cell carcinomas. Nature. 2015;517:576–82. PMID: 25631445. 10.1038/nature14129 PMC4311405 25631445

[5] Kumar M, Molkentine D, Molkentine J, Bridges K, Xie T, Yang L, et al. Inhibition of histone acetyltranserase function radiosensitizes CREBBP/EP300 mutants via repression of homologous recombination, potentially targeting a gain of function. Nat Commun. 2021;12(1):6340. 10.1038/s41467-021-26570-8. 10.1038/s41467-021-26570-8 PMC8566594 34732714

[6] Uzunparmak B, Gao M, Lindemann A, Erikson K, Wang L, Lin E, et al. Caspase-8 loss radiosensitizes head and neck squamous cell carcinoma to SMAC mimetic-induced necroptosis. JCI Insight. 2020;5(23):e139837. 10.1172/jci.insight.139837. 10.1172/jci.insight.139837 PMC7714407 33108350

[7] Neskey DM, Osman AA, Ow TJ, Katsonis P, McDonald T, Hicks SC, Hsu TK, Pickering CR, Ward A, Patel A (2015). Evolutionary action score of TP53 identifies high-risk mutations associated with decreased survival and increased distant metastases in head and neck Cancer. Cancer Res.

[8] Osman AA, Neskey DM, Katsonis P, Patel AA, Ward AM, Hsu TK, Hicks SC, McDonald TO, Ow TJ, Alves MO (2015). Evolutionary action score of TP53 coding variants is predictive of platinum response in head and neck Cancer patients. Cancer Res.

[9] Skinner HD, Sandulache VC, Ow TJ, Meyn RE, Yordy JS, Beadle BM, Fitzgerald AL, Giri U, Ang KK, Myers JN (2012). TP53 disruptive mutations lead to head and neck cancer treatment failure through inhibition of radiation-induced senescence. Clin Cancer Res.

[10] Zhou G, Wang J, Zhao M, Xie TX, Tanaka N, Sano D, Patel AA, Ward AM, Sandulache VC, Jasser SA (2014). Gain-of-function mutant p53 promotes cell growth and cancer cell metabolism via inhibition of AMPK activation. Mol Cell.

[11] Abraham AG, O'Neill E (2014). PI3K/Akt-mediated regulation of p53 in cancer. Biochem Soc Trans.

[12] Du L, Shen J, Weems A, Lu SL (2012). Role of phosphatidylinositol-3-kinase pathway in head and neck squamous cell carcinoma. J Oncol.

[13] Perez Sayans M, Chamorro Petronacci CM, Lorenzo Pouso AI, Padin Iruegas E, Blanco Carrion A, Suarez Penaranda JM, Garcia A (2019). Comprehensive genomic review of TCGA head and neck squamous cell carcinomas (HNSCC). J Clin Med.

[14] Lee C, Kim J-S, Waldman T (2007). Activated PI3K signaling as an endogenous inducer of p53 in human cancer. Cell Cycle.

[15] Jung K, Kang H, Mehra R (2018). Targeting phosphoinositide 3-kinase (PI3K) in head and neck squamous cell carcinoma (HNSCC). Cancers Head Neck.

[16] Cai Y, Dodhia S, Su GH (2017). Dysregulations in the PI3K pathway and targeted therapies for head and neck squamous cell carcinoma. Oncotarget.

[17] Schuler PJ, Harasymczuk M, Visus C, Deleo A, Trivedi S, Lei Y, Argiris A, Gooding W, Butterfield LH, Whiteside TL, Ferris RL (2014). Phase I dendritic cell p53 peptide vaccine for head and neck cancer. Clin Cancer Res.

[18] Biktasova A, Hajek M, Sewell A, Gary C, Bellinger G, Deshpande HA, Bhatia A, Burtness B, Judson B, Mehra S (2017). Demethylation therapy as a targeted treatment for human papillomavirus-associated head and neck Cancer. Clin Cancer Res.

[19] Castellanos MR, Pan Q (2016). Novel p53 therapies for head and neck cancer. World J Otorhinolaryngol Head Neck Surg.

[20] Zhou G, Liu Z, Myers JN (2016). TP53 mutations in head and neck squamous cell carcinoma and their impact on disease progression and treatment response. J Cell Biochem.

[21] Ota I, Okamoto N, Yane K, Takahashi A, Masui T, Hosoi H, Ohnishi T (2012). Therapeutic strategies for head and neck cancer based on p53 status. Exp Ther Med.

[22] Chen SMY, Li B, Nicklawsky AG, Krinsky AL, Brunetti T, Woolaver RA, Wang X, Chen Z, Young CD, Gao D (2020). Deletion of p53 and hyper-activation of PIK3CA in Keratin-15(+) stem cells Lead to the development of spontaneous squamous cell carcinoma. Int J Mol Sci.

[23] Du L, Chen X, Cao Y, Lu L, Zhang F, Bornstein S, Li Y, Owens P, Malkoski S, Said S (2016). Overexpression of PIK3CA in murine head and neck epithelium drives tumor invasion and metastasis through PDK1 and enhanced TGFβ signaling. Oncogene.

[24] Savar A, Acin S, Gonzalez CL, El-Sawy T, Mejia O, Li Z, Esmaeli B, Lacy-Hulbert A, El-Naggar AK, McCarty JH, Caulin C (2015). Loss of epithelial p53 and αv integrin cooperate through Akt to induce squamous cell carcinoma yet prevent remodeling of the tumor microenvironment. Oncogene.

[25] García-Carracedo D, Cai Y, Qiu W, Saeki K, Friedman RA, Lee A, Li Y, Goldberg EM, Stratikopoulos EE, Parsons R (2020). PIK3CA and p53 mutations promote 4NQO-Initated head and neck tumor progression and metastasis in mice. Mol Cancer Res.

[26] Chen SMY, Krinsky AL, Woolaver RA, Wang X, Chen Z, Wang JH (2020). Tumor immune microenvironment in head and neck cancers. Mol Carcinog.

[27] Bronte V, Brandau S, Chen SH, Colombo MP, Frey AB, Greten TF, Mandruzzato S, Murray PJ, Ochoa A, Ostrand-Rosenberg S (2016). Recommendations for myeloid-derived suppressor cell nomenclature and characterization standards. Nat Commun.

[28] Li B, Ren M, Zhou X, Han Q, Cheng L (2020). Targeting tumor-associated macrophages in head and neck squamous cell carcinoma. Oral Oncol.

[29] Boutilier AJ, Elsawa SF (2021). Macrophage polarization states in the tumor microenvironment. Int J Mol Sci.

[30] Costa NL, Valadares MC, Souza PP, Mendonça EF, Oliveira JC, Silva TA, Batista AC (2013). Tumor-associated macrophages and the profile of inflammatory cytokines in oral squamous cell carcinoma. Oral Oncol.

[31] Seiwert TY, Burtness B, Mehra R, Weiss J, Berger R, Eder JP, Heath K, McClanahan T, Lunceford J, Gause C (2016). Safety and clinical activity of pembrolizumab for treatment of recurrent or metastatic squamous cell carcinoma of the head and neck (KEYNOTE-012): an open-label, multicentre, phase 1b trial. Lancet Oncol.

[32] Ferris RL, Blumenschein G, Fayette J, Guigay J, Colevas AD, Licitra L, Harrington K, Kasper S, Vokes EE, Even C (2016). Nivolumab for recurrent squamous-cell carcinoma of the head and neck. N Engl J Med.

[33] Mehra R, Seiwert TY, Gupta S, Weiss J, Gluck I, Eder JP, Burtness B, Tahara M, Keam B, Kang H (2018). Efficacy and safety of pembrolizumab in recurrent/metastatic head and neck squamous cell carcinoma: pooled analyses after long-term follow-up in KEYNOTE-012. Br J Cancer.

[34] Hanna GJ, Lizotte P, Cavanaugh M, Kuo FC, Shivdasani P, Frieden A, Chau NG, Schoenfeld JD, Lorch JH, Uppaluri R (2018). Frameshift events predict anti-PD-1/L1 response in head and neck cancer. JCI Insight.

[35] Ferris RL, Blumenschein G, Fayette J, Guigay J, Colevas AD, Licitra L, Harrington KJ, Kasper S, Vokes EE, Even C (2018). Nivolumab vs investigator's choice in recurrent or metastatic squamous cell carcinoma of the head and neck: 2-year long-term survival update of CheckMate 141 with analyses by tumor PD-L1 expression. Oral Oncol.

[36] Harrington KJ, Ferris RL, Blumenschein G, Colevas AD, Fayette J, Licitra L, Kasper S, Even C, Vokes EE, Worden F (2017). Nivolumab versus standard, single-agent therapy of investigator's choice in recurrent or metastatic squamous cell carcinoma of the head and neck (CheckMate 141): health-related quality-of-life results from a randomised, phase 3 trial. Lancet Oncol.

[37] Ran X, Yang K (2017). Inhibitors of the PD-1/PD-L1 axis for the treatment of head and neck cancer: current status and future perspectives. Drug Des Devel Ther.

[38] Chow LQM, Haddad R, Gupta S, Mahipal A, Mehra R, Tahara M, Berger R, Eder JP, Burtness B, Lee SH (2016). Antitumor activity of Pembrolizumab in biomarker-unselected patients with recurrent and/or metastatic head and neck squamous cell carcinoma: results from the phase Ib KEYNOTE-012 expansion cohort. J Clin Oncol.

[39] Wagner M, Koester H, Deffge C, Weinert S, Lauf J, Francke A, et al. Isolation and intravenous injection of murine bone marrow derived monocytes. J Vis Exp. 2014;(94):52347. 10.3791/52347. 10.3791/52347 PMC4354490 25591000

[40] Zhu A, Ibrahim JG, Love MI (2019). Heavy-tailed prior distributions for sequence count data: removing the noise and preserving large differences. Bioinformatics.

[41] Li H, Durbin R (2009). Fast and accurate short read alignment with burrows-wheeler transform. Bioinformatics.

[42] Li H (2011). A statistical framework for SNP calling, mutation discovery, association mapping and population genetical parameter estimation from sequencing data. Bioinformatics.

[43] Cingolani P, Platts A, Wang le L, Coon M, Nguyen T, Wang L, Land SJ, Lu X, Ruden DM (2012). A program for annotating and predicting the effects of single nucleotide polymorphisms, SnpEff: SNPs in the genome of Drosophila melanogaster strain w1118; iso-2; iso-3. Fly (Austin).

[44] Cao Y, Wang X, Peng G (2020). SCSA: a cell type annotation tool for single-cell RNA-seq data. Front Genet.

[45] Franzén O, Gan LM, Björkegren JLM (2019). PanglaoDB: a web server for exploration of mouse and human single-cell RNA sequencing data. Database (Oxford).

[46] Menke J, Kriegsmann J, Schimanski CC, Schwartz MM, Schwarting A, Kelley VR (2012). Autocrine CSF-1 and CSF-1 receptor coexpression promotes renal cell carcinoma growth. Cancer Res.

[47] Kuang W, Deng Q, Deng C, Li W, Shu S, Zhou M (2017). Hepatocyte growth factor induces breast cancer cell invasion via the PI3K/Akt and p38 MAPK signaling pathways to up-regulate the expression of COX2. Am J Transl Res.

[48] Owusu BY, Galemmo R, Janetka J, Klampfer L (2017). Hepatocyte growth factor, a key tumor-promoting factor in the tumor microenvironment. Cancers (Basel).

[49] Sleightholm RL, Neilsen BK, Li J, Steele MM, Singh RK, Hollingsworth MA, Oupicky D (2017). Emerging roles of the CXCL12/CXCR4 axis in pancreatic cancer progression and therapy. Pharmacol Ther.

[50] Pollard JW (2004). Tumour-educated macrophages promote tumour progression and metastasis. Nat Rev Cancer.

[51] Gabrilovich D (2004). Mechanisms and functional significance of tumour-induced dendritic-cell defects. Nat Rev Immunol.

[52] Masuda M, Ruan HY, Ito A, Nakashima T, Toh S, Wakasaki T, Yasumatsu R, Kutratomi Y, Komune S, Weinstein IB (2007). Signal transducers and activators of transcription 3 up-regulates vascular endothelial growth factor production and tumor angiogenesis in head and neck squamous cell carcinoma. Oral Oncol.

[53] Niu G, Wright KL, Huang M, Song L, Haura E, Turkson J, Zhang S, Wang T, Sinibaldi D, Coppola D (2002). Constitutive Stat3 activity up-regulates VEGF expression and tumor angiogenesis. Oncogene.

[54] Xie TX, Wei D, Liu M, Gao AC, Ali-Osman F, Sawaya R, Huang S (2004). Stat3 activation regulates the expression of matrix metalloproteinase-2 and tumor invasion and metastasis. Oncogene.

[55] Ruffell B, Affara NI, Coussens LM (2012). Differential macrophage programming in the tumor microenvironment. Trends Immunol.

[56] Linde N, Lederle W, Depner S, van Rooijen N, Gutschalk CM, Mueller MM (2012). Vascular endothelial growth factor-induced skin carcinogenesis depends on recruitment and alternative activation of macrophages. J Pathol.

[57] Sulpice E, Ding S, Muscatelli-Groux B, Bergé M, Han ZC, Plouet J, Tobelem G, Merkulova-Rainon T (2009). Cross-talk between the VEGF-A and HGF signalling pathways in endothelial cells. Biol Cell.

[58] Ma PC, Tretiakova MS, Nallasura V, Jagadeeswaran R, Husain AN, Salgia R (2007). Downstream signalling and specific inhibition of c-MET/HGF pathway in small cell lung cancer: implications for tumour invasion. Br J Cancer.

[59] Hsieh RW, Borson S, Tsagianni A, Zandberg DP (2021). Immunotherapy in recurrent/metastatic squamous cell carcinoma of the head and neck. Front Oncol.

[60] Borel C, Jung AC, Burgy M (2020). Immunotherapy breakthroughs in the treatment of recurrent or metastatic head and neck squamous cell carcinoma. Cancers (Basel).

[61] Grasso CS, Tsoi J, Onyshchenko M, Abril-Rodriguez G, Ross-Macdonald P, Wind-Rotolo M, Champhekar A, Medina E, Torrejon DY, Shin DS (2020). Conserved interferon-γ signaling drives clinical response to immune checkpoint blockade therapy in melanoma. Cancer Cell.

[62] Jorgovanovic D, Song M, Wang L, Zhang Y (2020). Roles of IFN-γ in tumor progression and regression: a review. Biomark Res.

[63] Ayers M, Lunceford J, Nebozhyn M, Murphy E, Loboda A, Kaufman DR, Albright A, Cheng JD, Kang SP, Shankaran V (2017). IFN-gamma-related mRNA profile predicts clinical response to PD-1 blockade. J Clin Invest.

[64] Woolaver RA, Wang X, Krinsky AL, Waschke BC, Chen SMY, Popolizio V, Nicklawsky AG, Gao D, Chen Z, Jimeno A (2021). Differences in TCR repertoire and T cell activation underlie the divergent outcomes of antitumor immune responses in tumor-eradicating versus tumor-progressing hosts. J Immunother Cancer.

[65] Petty AJ, Yang Y (2017). Tumor-associated macrophages: implications in cancer immunotherapy. Immunotherapy.

[66] Ge Z, Ding S (2020). The crosstalk between tumor-associated macrophages (TAMs) and tumor cells and the corresponding targeted therapy. Front Oncol.

[67] Yang H, Zhang Q, Xu M, Wang L, Chen X, Feng Y, Li Y, Zhang X, Cui W, Jia X (2020). CCL2-CCR2 axis recruits tumor associated macrophages to induce immune evasion through PD-1 signaling in esophageal carcinogenesis. Mol Cancer.

[68] Kumar AT, Knops A, Swendseid B, Martinez-Outschoom U, Harshyne L, Philp N, Rodeck U, Luginbuhl A, Cognetti D, Johnson J, Curry J (2019). Prognostic significance of tumor-associated macrophage content in head and neck squamous cell carcinoma: a Meta-analysis. Front Oncol.

[69] Van Overmeire E, Stijlemans B, Heymann F, Keirsse J, Morias Y, Elkrim Y, Brys L, Abels C, Lahmar Q, Ergen C (2016). M-CSF and GM-CSF receptor signaling differentially regulate monocyte maturation and macrophage polarization in the tumor microenvironment. Cancer Res.

[70] Chockalingam S, Ghosh SS (2014). Macrophage colony-stimulating factor and cancer: a review. Tumour Biol.

[71] De Palma M (2012). Partners in crime: VEGF and IL-4 conscript tumour-promoting macrophages. J Pathol.

[72] Guo XY, Zhang JY, Shi XZ, Wang Q, Shen WL, Zhu WW, Liu LK (2020). Upregulation of CSF-1 is correlated with elevated TAM infiltration and poor prognosis in oral squamous cell carcinoma. Am J Transl Res.

[73] Albert S, Riveiro ME, Halimi C, Hourseau M, Couvelard A, Serova M, Barry B, Raymond E, Faivre S (2013). Focus on the role of the CXCL12/CXCR4 chemokine axis in head and neck squamous cell carcinoma. Head Neck.

[74] Li Y, Wu T, Gong S, Zhou H, Yu L, Liang M, Shi R, Wu Z, Zhang J, Li S (2020). Analysis of the prognosis and therapeutic value of the CXC chemokine family in head and neck squamous cell carcinoma. Front Oncol.

[75] Liu D, Zhong M, Zhan D, Zhang Y, Liu S (2020). Roles of the HGF/met signaling in head and neck squamous cell carcinoma: focus on tumor immunity (review). Oncol Rep.

[76] Zhang YW, Wang LM, Jove R, Vande Woude GF (2002). Requirement of Stat3 signaling for HGF/SF-met mediated tumorigenesis. Oncogene.

[77] Syed ZA, Yin W, Hughes K, Gill JN, Shi R, Clifford JL (2011). HGF/c-met/Stat3 signaling during skin tumor cell invasion: indications for a positive feedback loop. BMC Cancer.

[78] Trovato M, Torre ML, Ragonese M, Simone A, Scarfì R, Barresi V, Giuffrè G, Benvenga S, Angileri FF, Tuccari G (2013). HGF/c-met system targeting PI3K/AKT and STAT3/phosphorylated-STAT3 pathways in pituitary adenomas: an immunohistochemical characterization in view of targeted therapies. Endocrine.

[79] Shi X, Kaller M, Rokavec M, Kirchner T, Horst D, Hermeking H (2020). Characterization of a p53/miR-34a/CSF1R/STAT3 feedback loop in colorectal Cancer. Cell Mol Gastroenterol Hepatol.

[80] Wang XY, Wang LL, Zheng X, Meng LN, Lyu B, Jin HF (2016). Expression of p-STAT3 and vascular endothelial growth factor in MNNG-induced precancerous lesions and gastric tumors in rats. World J Gastrointest Oncol.

[81] Johnson DE, O'Keefe RA, Grandis JR (2018). Targeting the IL-6/JAK/STAT3 signalling axis in cancer. Nat Rev Clin Oncol.

[82] Lei Y, Li X, Huang Q, Zheng X, Liu M (2021). Progress and challenges of predictive biomarkers for immune checkpoint blockade. Front Oncol.

[83] Korbecki J, Bajdak-Rusinek K, Kupnicka P, Kapczuk P, Simińska D, Chlubek D, Baranowska-Bosiacka I (2021). The role of CXCL16 in the pathogenesis of Cancer and other diseases. Int J Mol Sci.

[84] Murphy JP, Yu Q, Konda P, Paulo JA, Jedrychowski MP, Kowalewski DJ, Schuster H, Kim Y, Clements D, Jain A (2019). Multiplexed relative quantitation with isobaric tagging mass spectrometry reveals class I major histocompatibility complex ligand dynamics in response to doxorubicin. Anal Chem.

[85] Gaździcka J, Gołąbek K, Strzelczyk JK, Ostrowska Z (2020). Epigenetic modifications in head and neck Cancer. Biochem Genet.

[86] Bais MV (2019). Impact of epigenetic regulation on head and neck squamous cell carcinoma. J Dent Res.

[87] Cameron SR, Dahler AL, Endo-Munoz LB, Jabbar I, Thomas GP, Leo PJ, Poth K, Rickwood D, Guminski A, Saunders NA (2010). Tumor-initiating activity and tumor morphology of HNSCC is modulated by interactions between clonal variants within the tumor. Lab Investig.

[88] Kaseb HO, Fohrer-Ting H, Lewis DW, Lagasse E, Gollin SM (2016). Identification, expansion and characterization of cancer cells with stem cell properties from head and neck squamous cell carcinomas. Exp Cell Res.

