# Supplementary material for: Differential responses to immune checkpoint inhibitor dictated by pre-existing differential immune profiles in squamous cell carcinomas caused by same initial oncogenic drivers
Source: J Exp Clin Cancer Res. 2022 Apr 2;41:123. doi: 10.1186/s13046-022-02337-x (PMC8976353; doi:10.1186/s13046-022-02337-x)
Supplement: Supplementary file 1 — Additional file 1. [file 13046_2022_2337_MOESM1_ESM.docx]

**Supplemental Method**

**Gene Set Enrichment Analysis** (**GSEA)**

Low expressed genes were removed from the dataset if they had <10 reads. Reads were normalized and differential expression was calculated using the DESeq function in the DESeq2 R package (1). GSEA was performed using the full list of genes ranked by Log2(fold change) for the comparison between TAb2 tumor cells vs TCh3 tumor cells and the clusterProfiler R package (2) using gene sets from the KEGG Database (<https://www.genome.jp/kegg/pathway.html>). The bar graph was generated with the ggplot2 R packages (3). The Gene-Concept network plot and GSEA plot were generated with the enrichplot R package (<https://yulab-smu.top/biomedical-knowledge-mining-book/>). The pathway plots were generated with pathview R package (4).

**References:**

1. Love MI, Huber W, Anders S. Moderated estimation of fold change and dispersion for RNA-seq data with DESeq2. Genome Biol. 2014;15(12):550.

2. Yu G, Wang LG, Han Y, He QY. clusterProfiler: an R package for comparing biological themes among gene clusters. Omics. 2012;16(5):284-7.

3. Wickham H. ggplot2: Elegant Graphics for Data Analysis. 2 ed. New York: Springer International Publishing; 2016. XVI, 260 p.

4. Luo W, Brouwer C. Pathview: an R/Bioconductor package for pathway-based data integration and visualization. Bioinformatics. 2013;29(14):1830-1.
