# Supplementary material for: Differential responses to immune checkpoint inhibitor dictated by pre-existing differential immune profiles in squamous cell carcinomas caused by same initial oncogenic drivers
Source: J Exp Clin Cancer Res. 2022 Apr 2;41:123. doi: 10.1186/s13046-022-02337-x (PMC8976353; doi:10.1186/s13046-022-02337-x)
Supplement: Supplementary file 2 — Additional file 2: Figure S1. Generation of daughter cell lines, western blotting and different tumor growth pattern of TAb2 vs. TCh3 tumors. Figure S2. Gating strategy for flow cytometry analysis. Figure S3. Differential expression of epigenetic modulators in TAb2 vs. TCh3 tumor cells. Figure S4. WES data of TAb2 vs TCh3 tumor cells. Figure S5. Growth curves of different myeloid populations upon co-culture of BM cells with TAb2 or TCh3 tumor cells. Figure S6. Gene Set Enrichment Analysis (GSEA) using KEGG pathway depicts transcriptional profiles of negatively or positively enriched in TAb2 and TCh3 tumor cells. Figure S7. Survival curves for HNSCC patients expressing different levels of CSF1 and/or VEGF. Figure S8. Flow cytometry analysis confirmed that anti-PD-L1 treatment did not affect the cell types present in the TME of TAb2 tumors and tumor-infiltrating CD8 T cell function compared to control TAb2 tumors. Figure S9. Detailed p-values for figures. [file 13046_2022_2337_MOESM2_ESM.pdf]

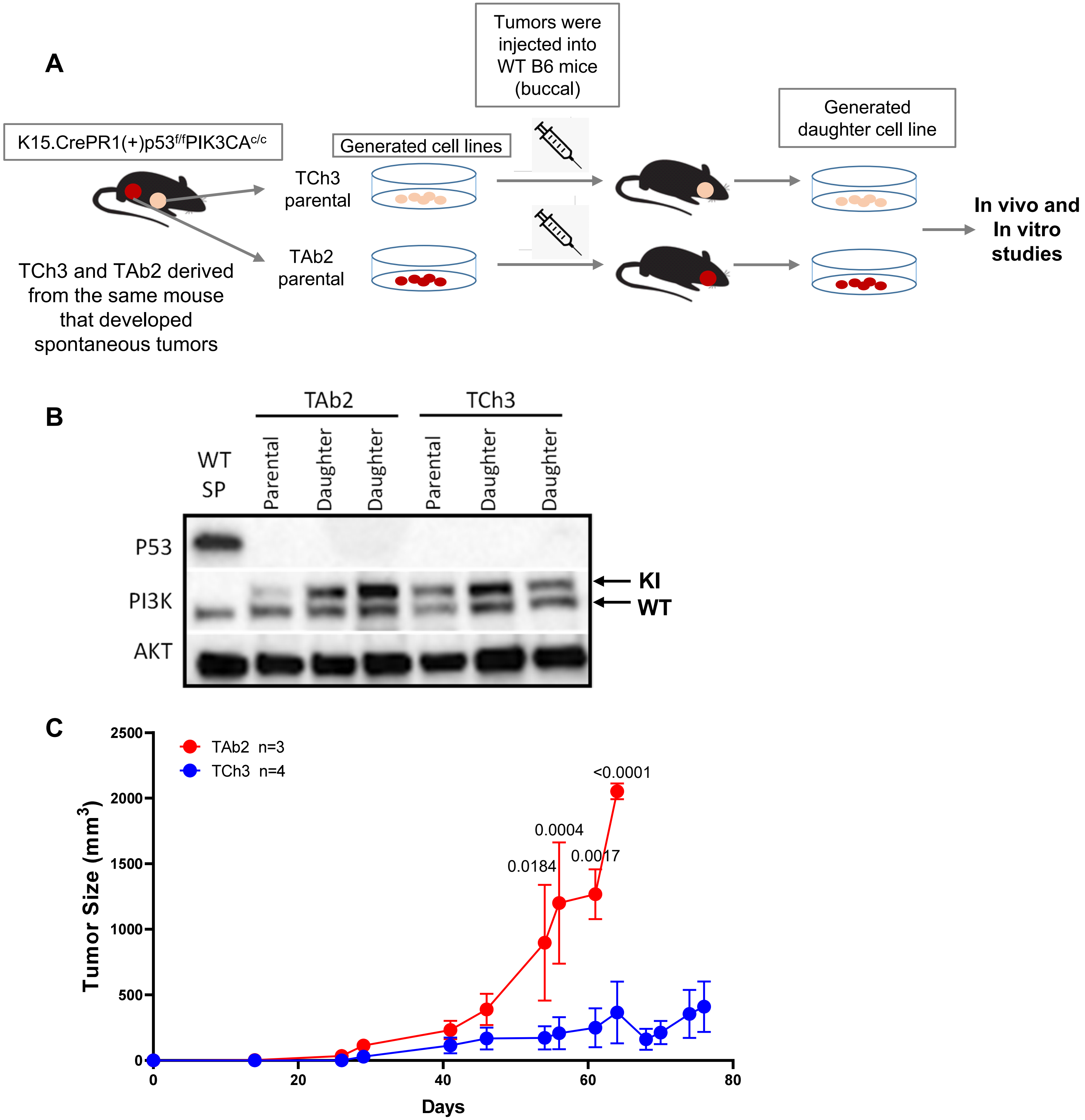

Figure S1. Establishment of TAb2 and TCh3 models. (A) Schematic diagram depicting the generation of KPPA tumor cell lines. **(B)** Western blotting data showing the absence of TP53 protein and the presence of PIK3CA hyperactive allele (KI) that encodes a protein slightly larger than WT PIK3CA protein (WT) in TAb2 and TCh3 parental and daughter cell lines. AKT as loading control. **(C)** Different tumor growth pattern of TAb2 versus TCh3 tumors. Tumor growth curves for TAb2 (n=3) and TCh3 (n=4) tumors when 1×10^6 tumor cells were injected subcutaneously at the flank of WT B6 mice. *P* values are shown for Sidak's multiple comparisons by two-way ANOVA.

Figure S1A-C

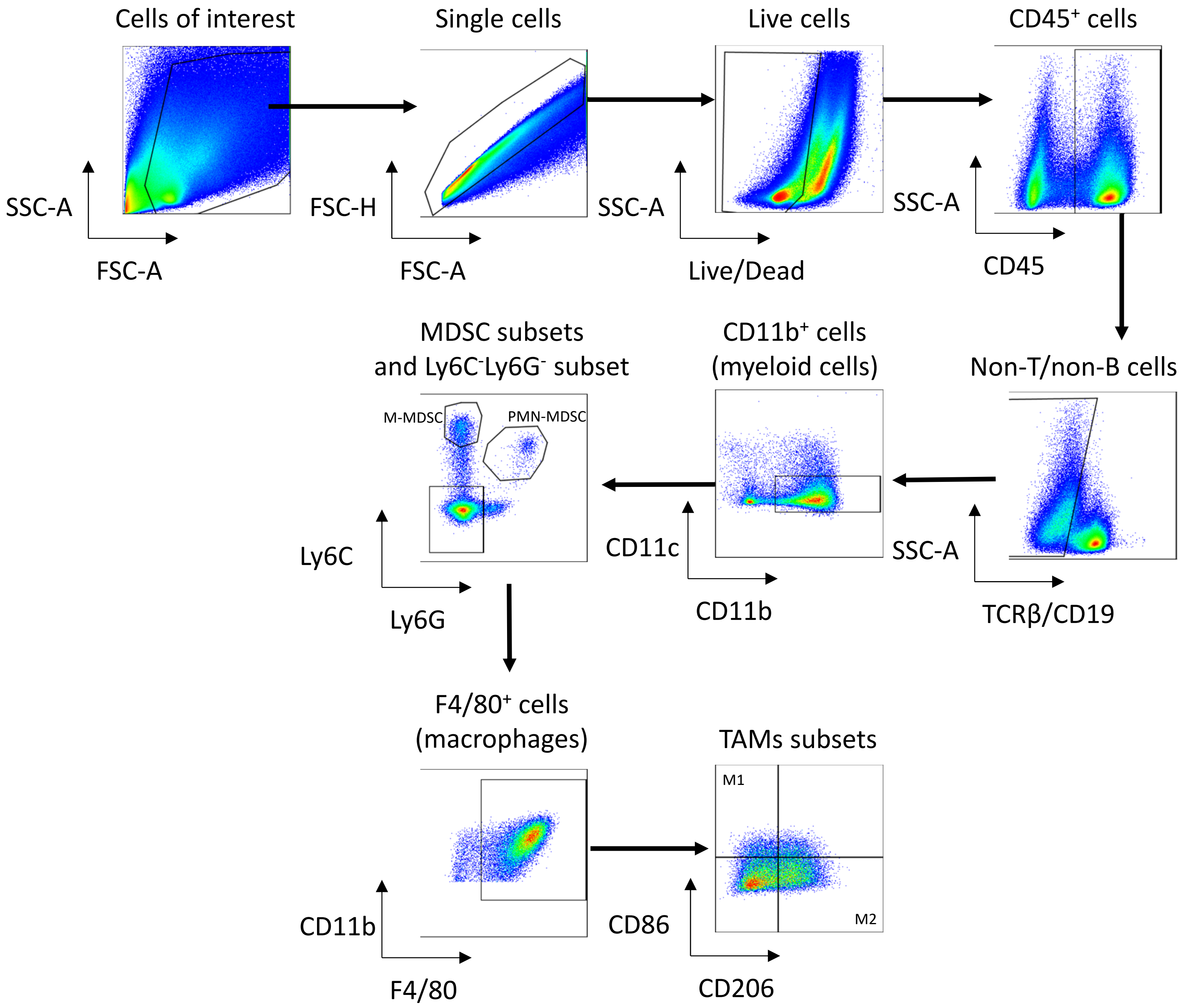

Figure S2. Gating strategy for different subsets of myeloid cells. After gating on CD45^{+} population, we gated on non-T/non-B population (TCRβ^{-}CD19^{-}). From the non-T/non-B population, we gated on CD11b^{+} population. In the CD11b^{+} population, we identified M-MDSC (Ly6C^{high}Ly6G^{-}) and PMN-MDSC (Ly6C^{low}Ly6G^{+}). For TAMs, we gated on Ly6C^{-}Ly6G^{-} population, then gated on F4/80^{+}CD11b^{+} population. For M2-TAMs, we gated on F4/80^{+}CD11b^{+}CD206^{+}CD86^{-} population.

Figure S2

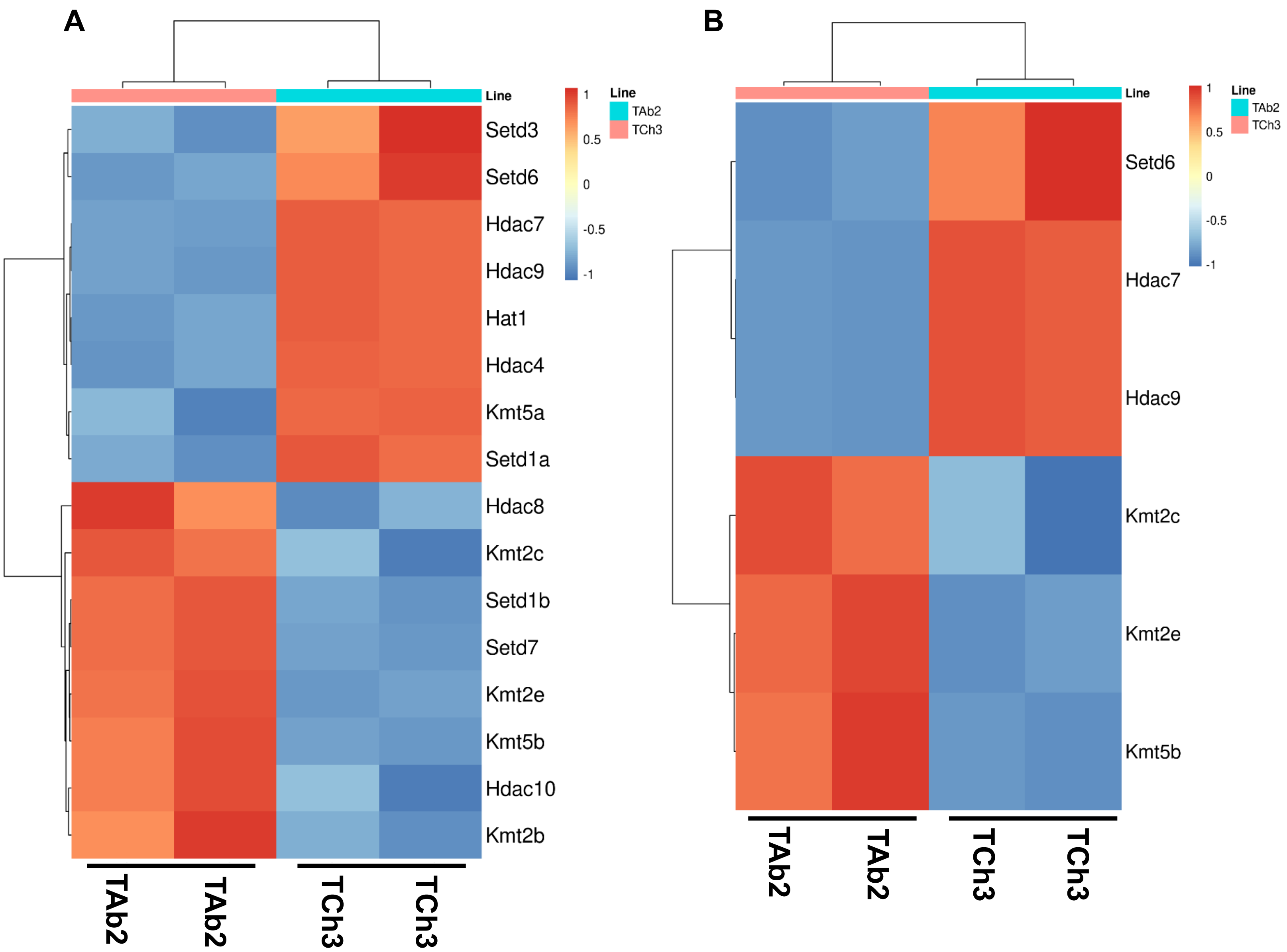

Figure S3. Heatmap of gene expression of selected epigenetic modulators in bulk RNA-seq data. Expression values for each gene are scaled across TAb2 (n=2) and TCh3 (n=2) tumor cells. Genes were filtered for those differentially expressed with a threshold of log2(fold change)=0.58 (1.5-fold difference) **(A)** or log2(fold change)=1 (2-fold difference) **(B)** and BH adjusted p-value=0.05.

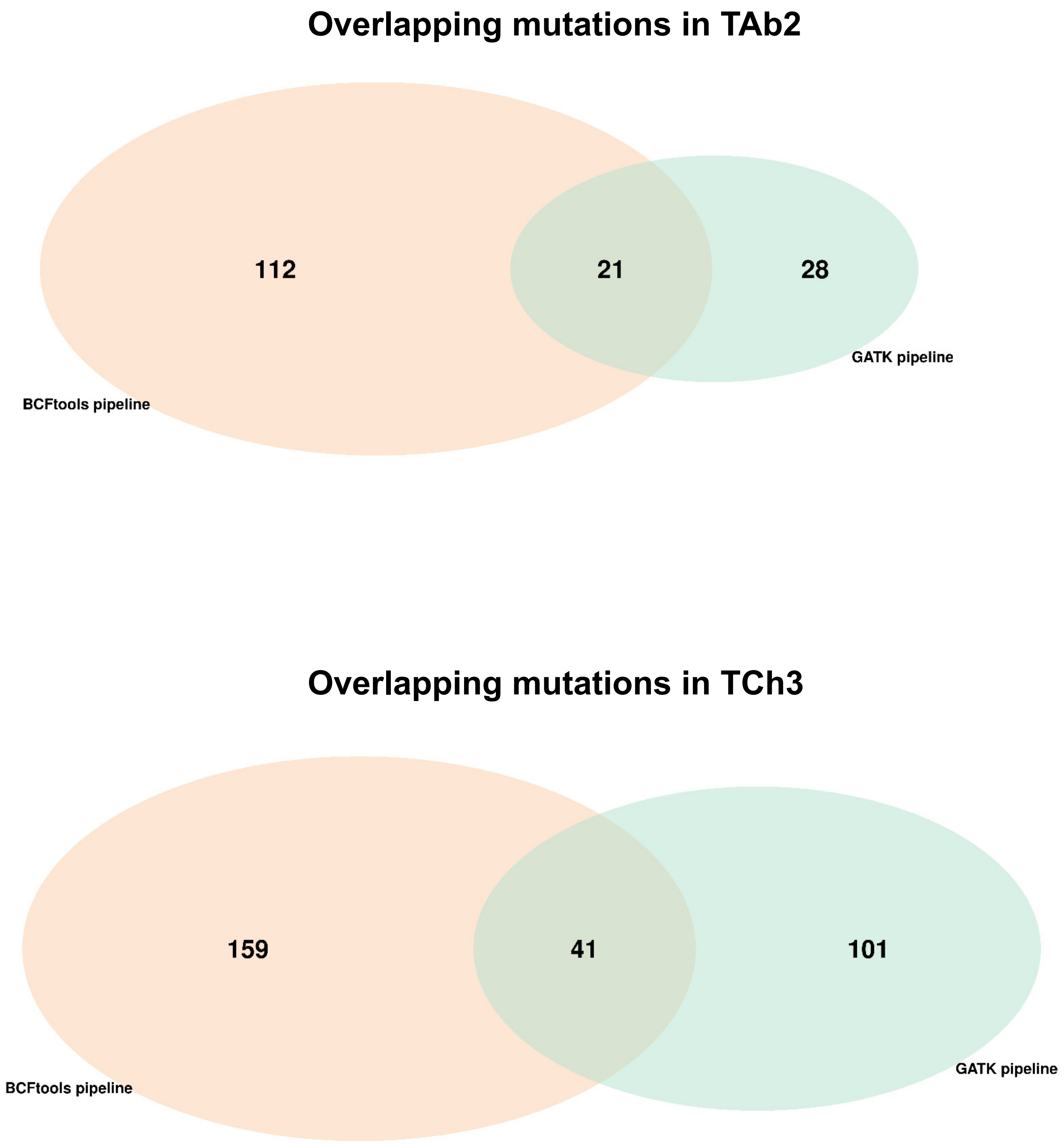

Figure S4. Somatic mutations identified in TAb2 and TCh3 tumors using both variant calling pipelines. Venn diagrams of overlapping mutations in TAb2 (top) and TCh3 (bottom) identified by GATK (right, light green) and BCFtools (left, light orange) pipelines in WES data (see details in Method).

Figure S4

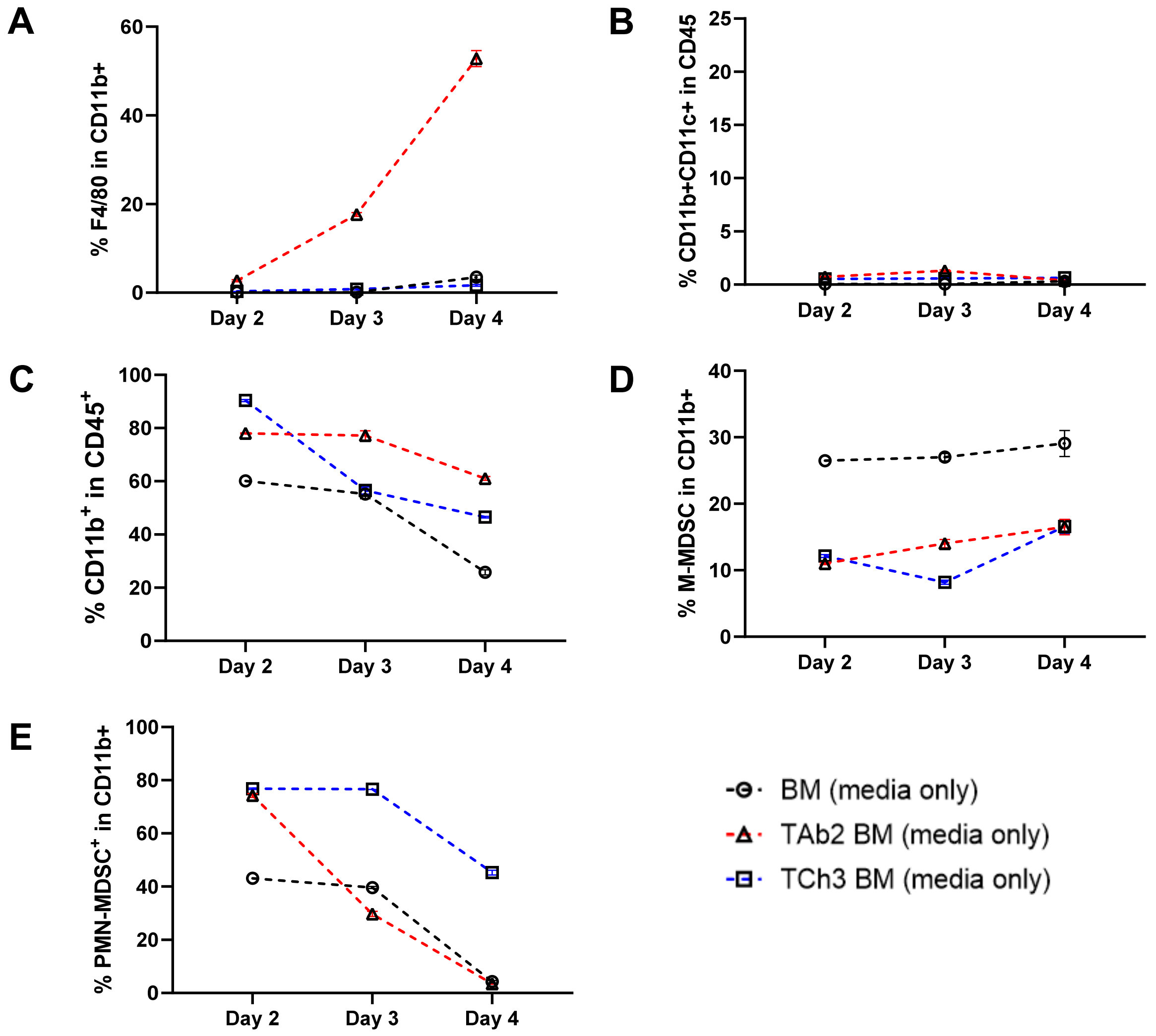

Figure S5. Growth curves of different myeloid populations upon co-culture of BM cells with TAb2 or TCh3 tumor cells. The frequencies of each cell type were determined by flow cytometry at different time points of co-culture (Day 2, 3 and 4). The frequency of **(A)** TAMs ($CD11b^+Ly6C^-Ly6G^-F4/80^+$), **(B)** dendritic cells ($CD11b^+CD11c^+$), **(C)** myeloid cells ($CD11b^+$), **(D)** M-MDSC ($CD11b^+Ly6G^-Ly6C^{hi}$), and **(E)** PMN-MDSC ($CD11b^+Ly6G^+Ly6C^{low}$). BM alone (black), TAb2-BM (red) and TCh3-BM (blue) co-culture are shown. Results are representative of more than three independent experiments done in triplicates.

A

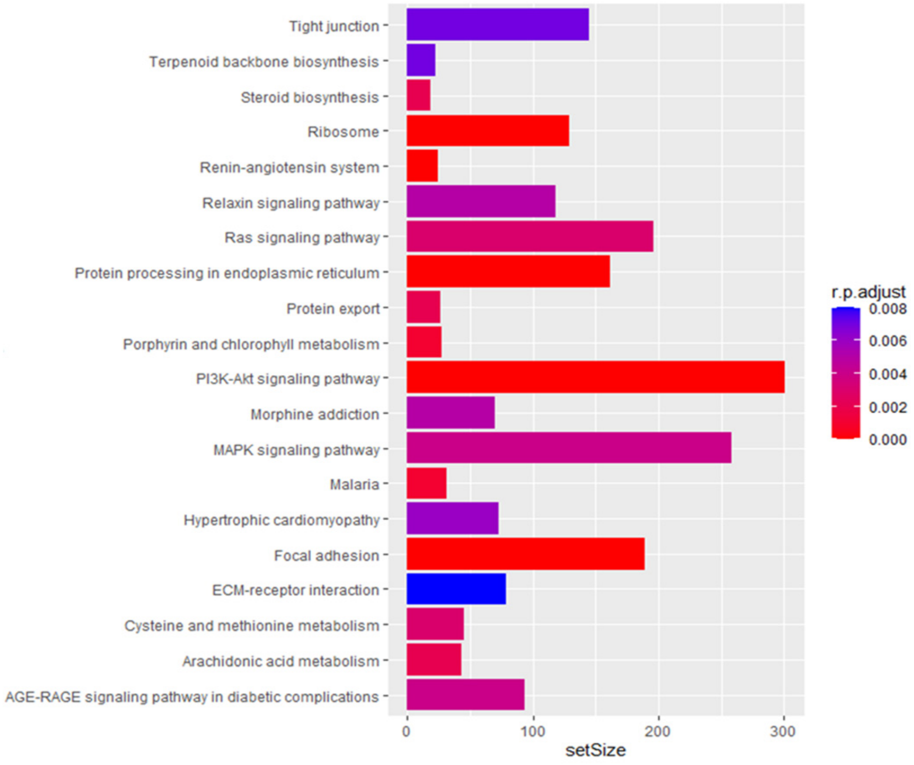

B

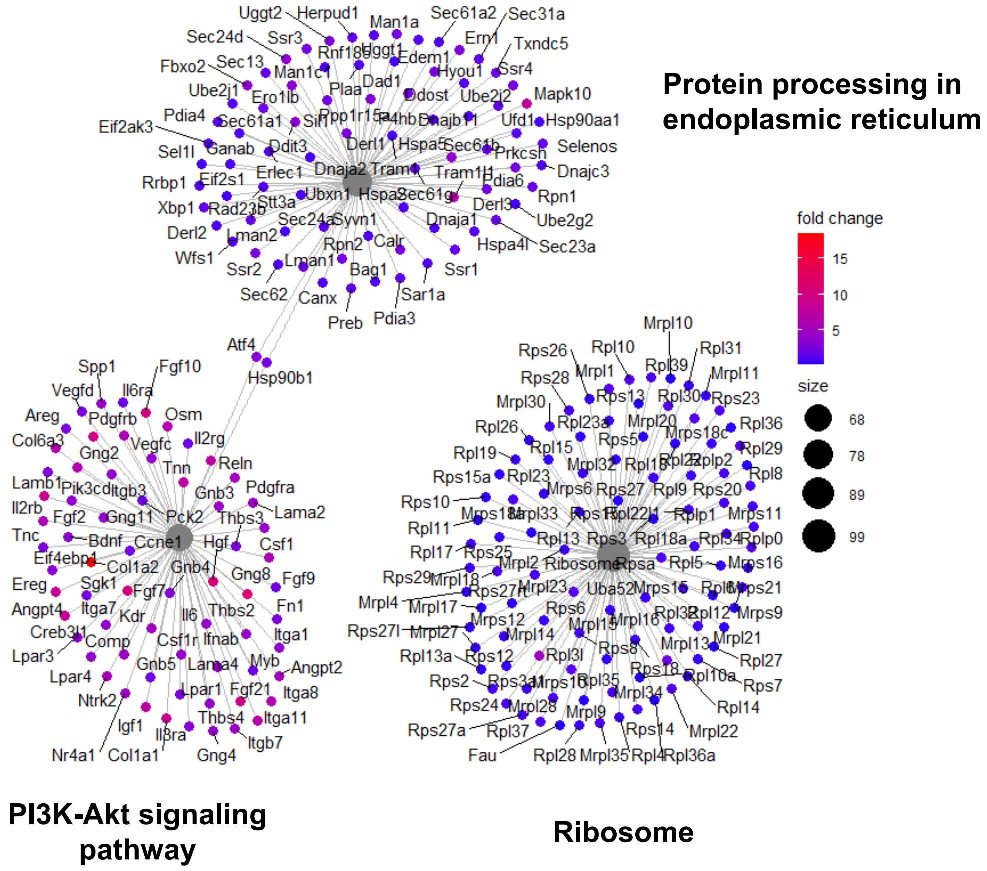

Figure S6A, B

C

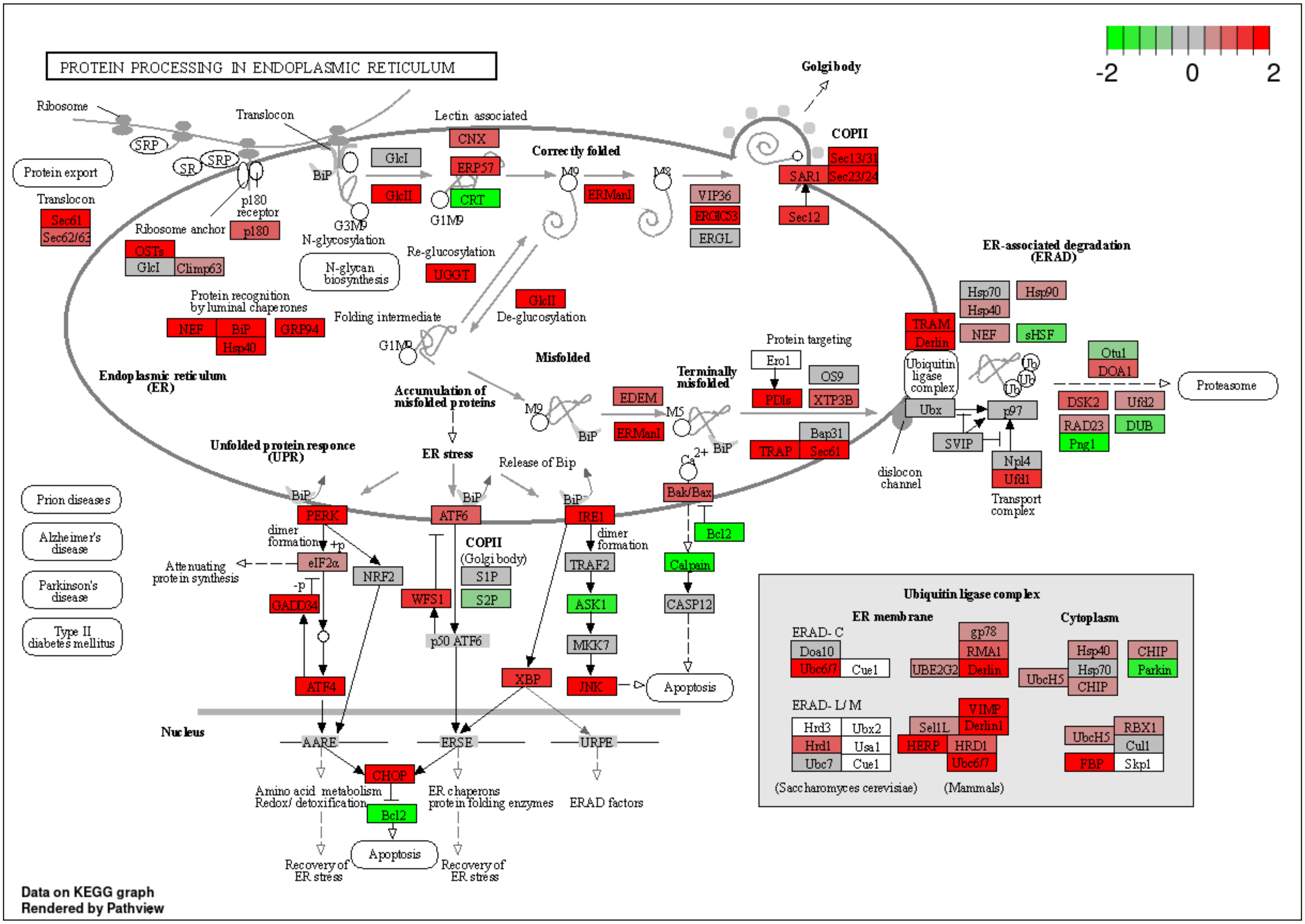

D

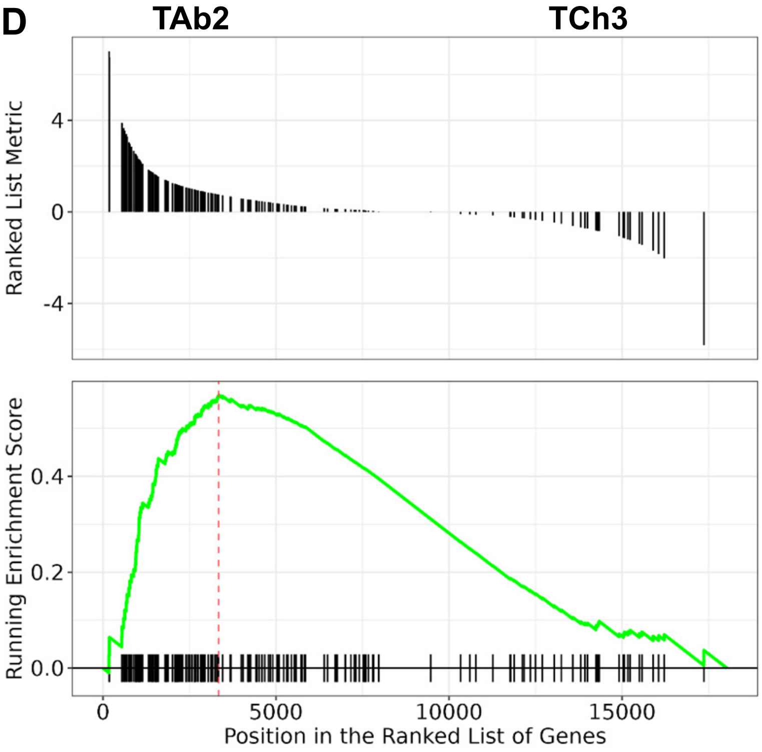

Figure S6C, D

E

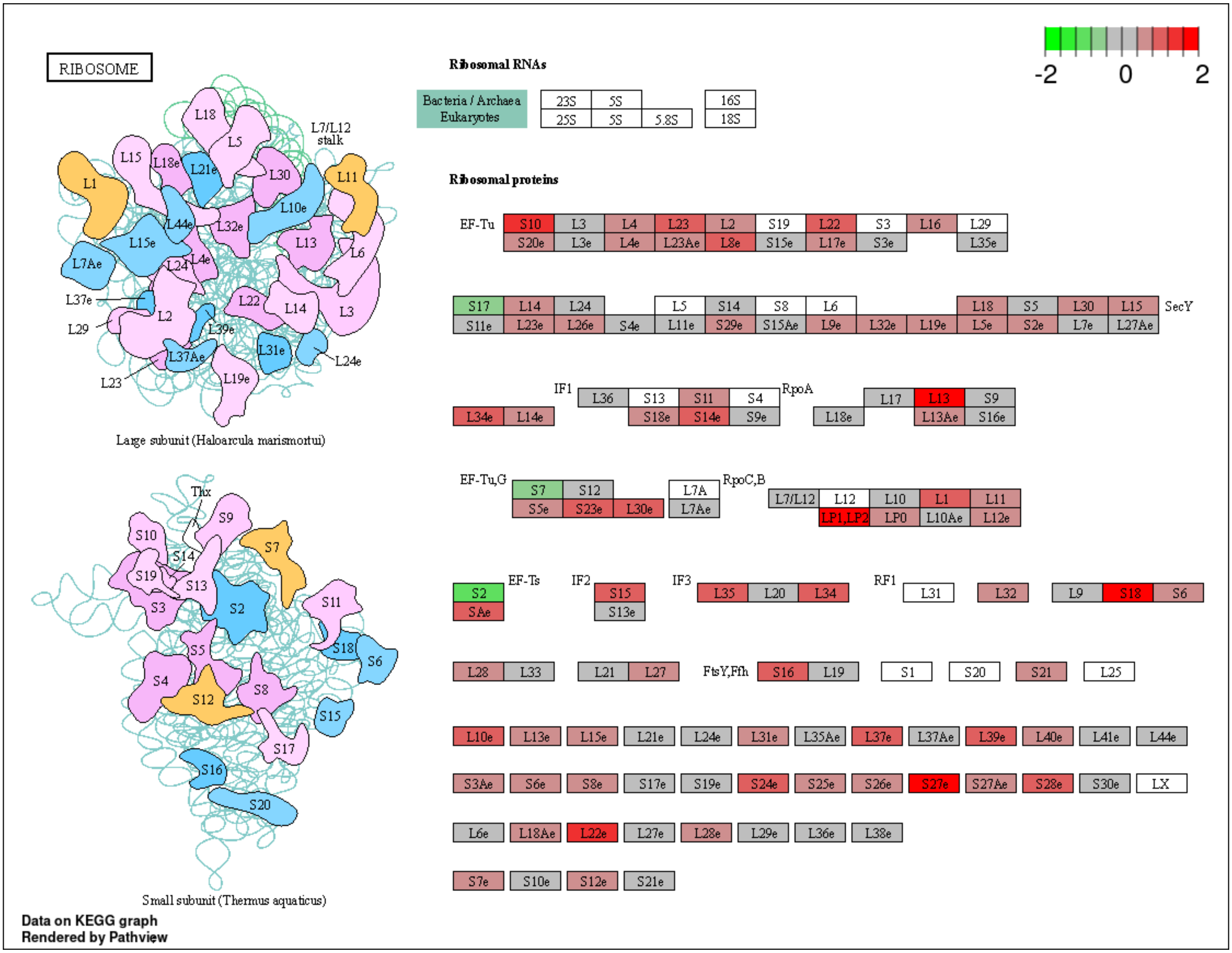

F

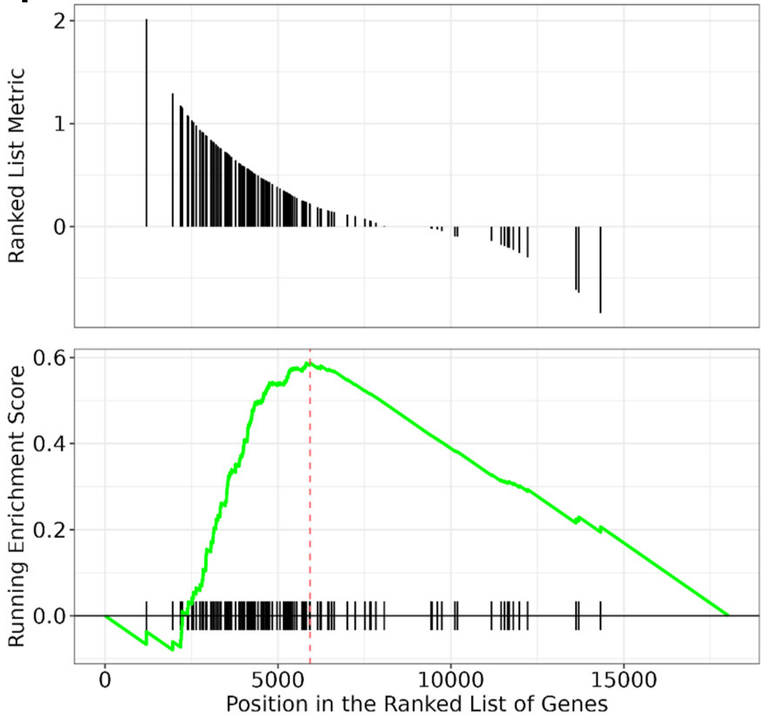

Figure S6E, F

G

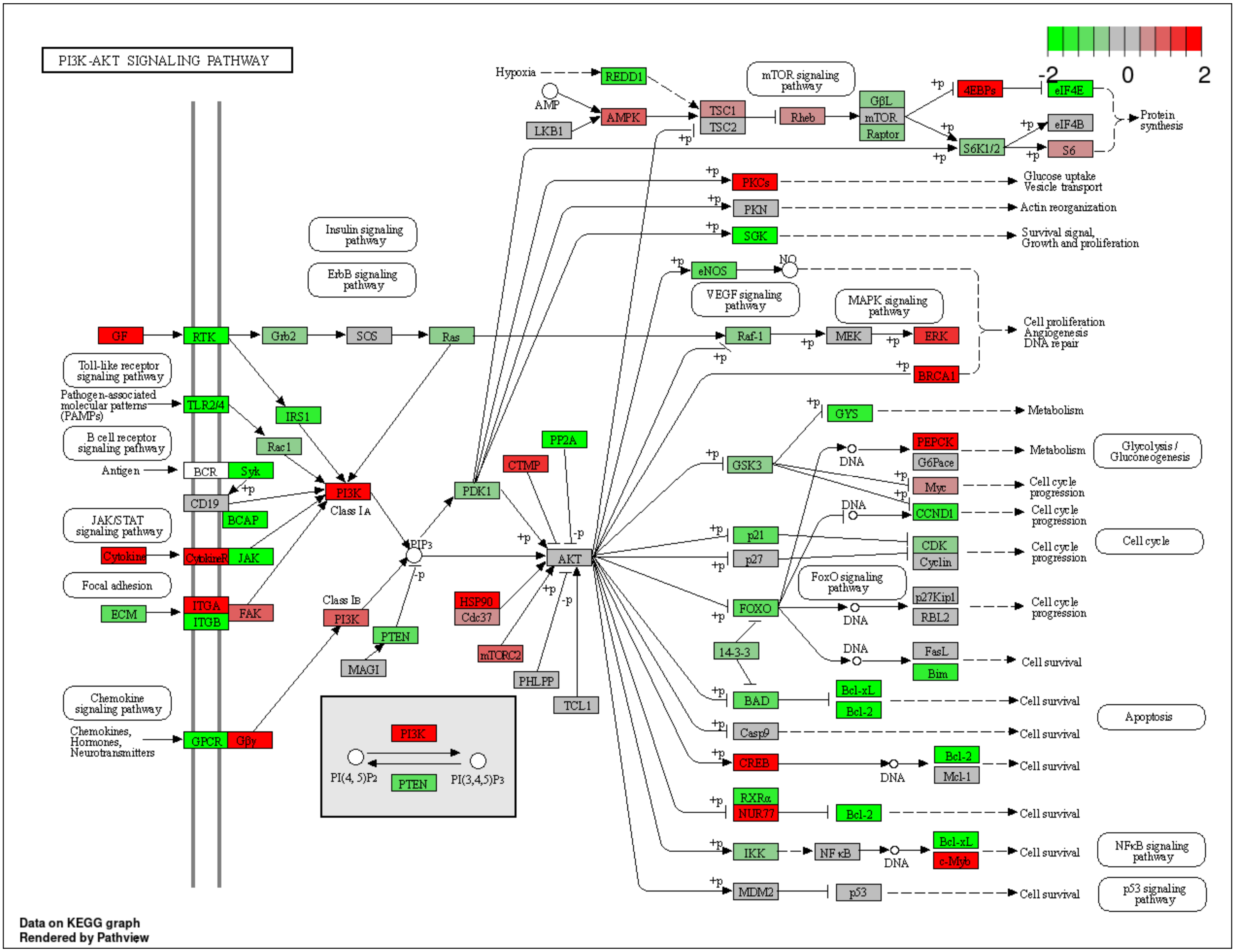

H

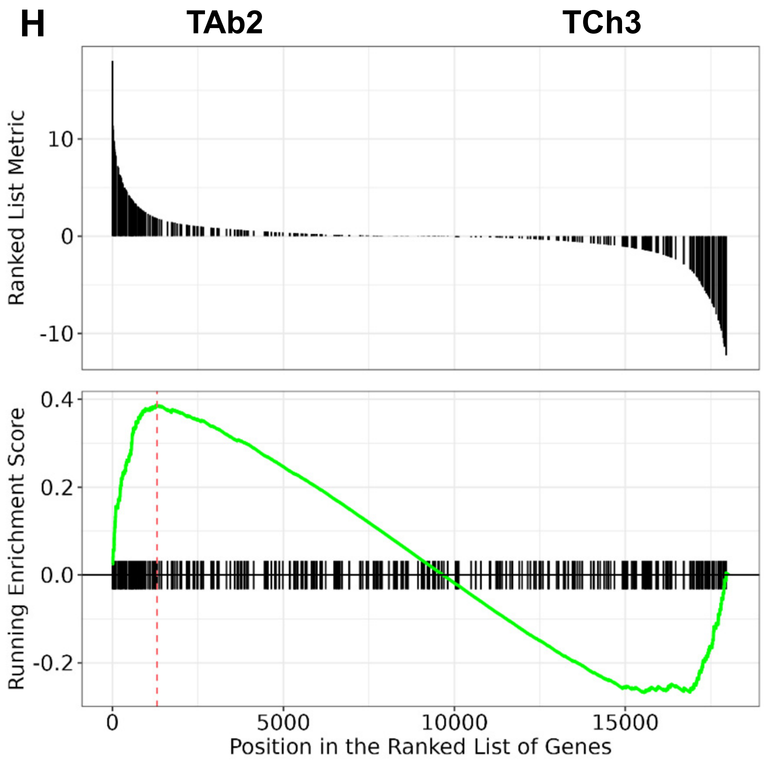

Figure S6G, H

Figure S6. Gene Set Enrichment Analysis (GSEA) using KEGG pathway depicts transcriptional profiles of negatively or positively enriched in TAb2 and TCh3 tumor cells. (A) The bar graph shows top 20 enriched KEGG pathways labeled on the y-axis with gene count (number of genes in the specific pathway from the output data of DESeq) on the x-axis and color-coded according to the adjusted p-value. **(B)** The Gene-Concept network plot shows the genes that are involved in the top three most significant pathways. The Gene-Concept Network depicts the linkages of genes and biological concepts as a network. The pathway circle size corresponds to the number of genes, while the genes themselves are color coded to reflect the fold change. **(C-H)** GSEA plots showing enrichment of protein processing in endoplasmic reticulum **(C, D)**, ribosome **(E, F)**, and PI3K-Akt signaling pathway **(G, H)**. To further assess the three most significant pathways, genes involved in each pathway is shown (C, E, and G). An enrichment score is calculated which represents the degrees to which a set of genes is over-represented at the top or bottom of the ranked list (D, F, and H). The green curve corresponds to the ES (enrichment score) curve, which is the running sum of the weighted ES obtained from GSEA software.

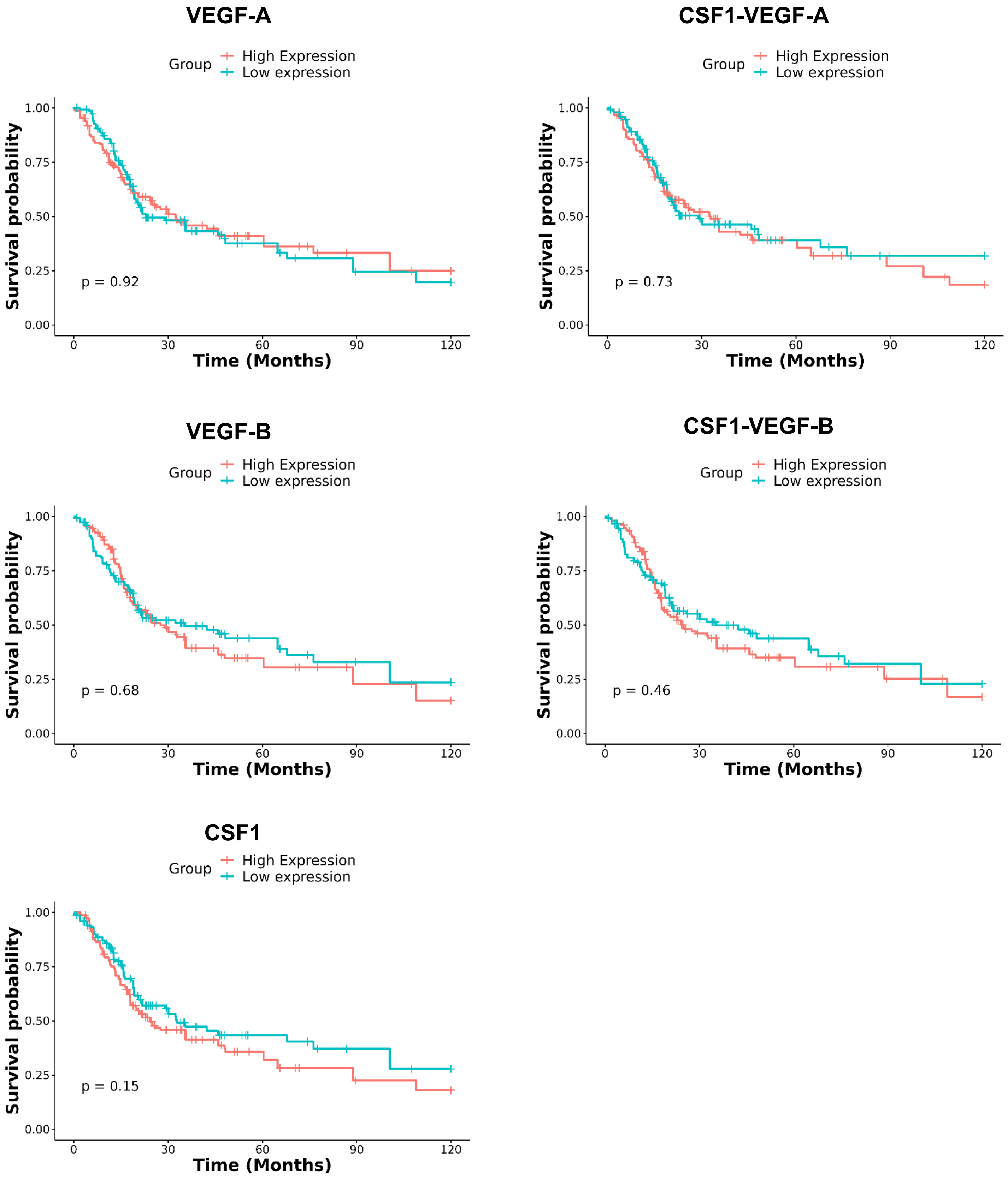

Figure S7. Survival curves for HNSCC patients expressing different levels of CSF1 and/or VEGF. 10-year survival Kaplan-Meier plots of HNSCC patients who had both PIK3CA^{Amp} and TP53^{Mutated} (n = 300). Patients were grouped into high-expression group or low-expression group based on gene expression as described in Methods.

Figure S7

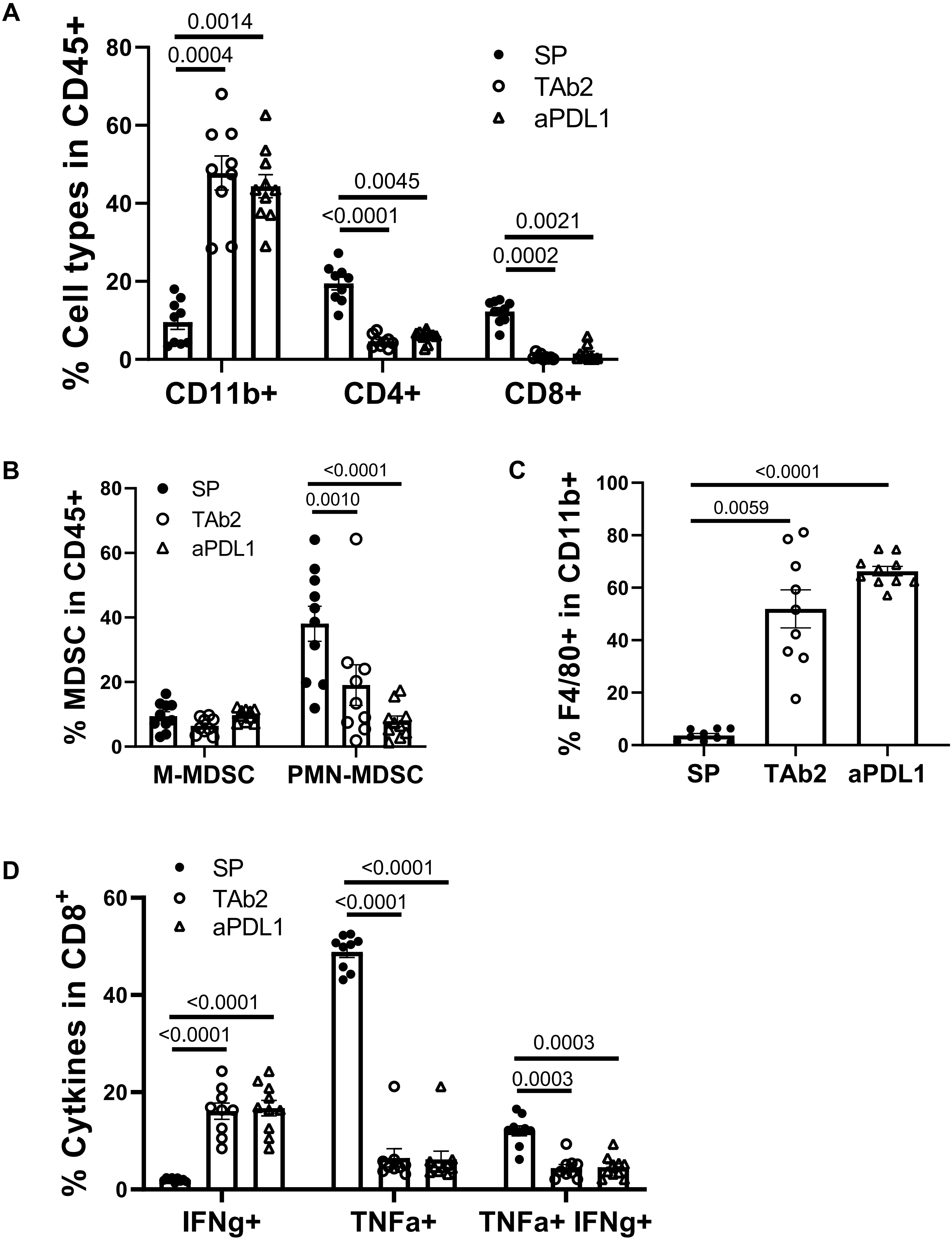
A
% Cell types in CD45+
0.0014
0.0004
0.0045
<0.0001
0.0021
0.0002
SP
TAb2
aPDL1
80
60
40
20
0
CD11b+
CD4+
CD8+
B
% MDSC in CD45+
<0.0001
0.0010
SP
TAb2
aPDL1
80
60
40
20
0
M-MDSC
PMN-MDSC
C
% F4/80+ in CD11b+
<0.0001
0.0059
100
80
60
40
20
0
SP
TAb2
aPDL1
D
% Cytkines in CD8+
SP
TAb2
aPDL1
<0.0001
<0.0001
<0.0001
<0.0001
0.0003
0.0003
60
40
20
0
IFNg+
TNFa+
TNFa+ IFNg+

Figure S8

Figure S8. Flow cytometry analysis confirmed that anti-PD-L1 treatment did not affect the cell types present in the TME of TAb2 tumors and tumor-infiltrating CD8 T cell function compared to control TAb2 tumors. Flow cytometry was performed for spleens (n=9), or the tumor-infiltrating immune cells from TAb2 VC (n=9) and TAb2 anti-PD-L1 (n=10) tumors for all panels. TAb2 tumor cells (0.5×10^6) were injected s.c., and tumors were harvested on Day 21 post-injection. **(A)** Quantification of the percentage of $CD11b^+$, $CD4^+$, or $CD8^+$ cells in $CD45^+$ population. Statistical significance was calculated using two-way ANOVA with Tukey's multiple comparison test. **(B)** Quantification of the percentages of MDSCs. M-MDSC ($CD11b^+Ly6G^-Ly6C^{high}$) and PMN-MDSC ($CD11b^+Ly6G^+Ly6C^{low}$). **(C)** Quantification of the percentages of TAMs ($CD11b^+Ly6G^-Ly6C^-F4/80^+$) in spleen, TAb2 control and TAb2-anti-PD-L1 treated tumors. *P* values are shown for Tukey's multiple comparisons by two-way ANOVA (MDSCs) and one-way ANOVA (TAMs). **(D)** Frequencies of the $CD8^+$ T cells producing single or double cytokines ($IFN\gamma^+$, $TNF\alpha^+$, and $IFN\gamma^+TNF\alpha^+$) in response to *ex vivo* stimulation. *P* values are shown for Tukey's multiple comparisons by two-way ANOVA.

A

Figure 2A

CD11b$^+$	P-values
SPLEEN (TCh3) vs. TAb2	<0.0001
SPLEEN (TCh3) vs. TCh3	<0.0001
TAb2 vs. TCh3	0.0053

CD4$^+$	P-values
SPLEEN (TCh3) vs. TAb2	0.0034
SPLEEN (TCh3) vs. TCh3	>0.9999
TAb2 vs. TCh3	0.0030

CD8$^+$	P-values
SPLEEN (TCh3) vs. TAb2	0.0024
SPLEEN (TCh3) vs. TCh3	>0.9999
TAb2 vs. TCh3	0.0091

Figure 2C

M-MDSC	P-values
Spleen vs. TAb2	0.7025
Spleen vs. TCh3	0.1298
TAb2 vs. TCh3	0.4346

PNM-MDSC	P-values
Spleen vs. TAb2	0.038
Spleen vs. TCh3	0.5895
TAb2 vs. TCh3	0.2843

F4/80	P-values
Spleen vs. TAb2	<0.0001
Spleen vs. TCh3	0.4327
TAb2 vs. TCh3	<0.0001

Figure 2E

CD206$^-$CD86$^+$	P-values
Spleen vs. TAb2	0.9581
Spleen vs. TCh3	0.1414
TAb2 vs. TCh3	0.2111

CD206$^+$CD86$^-$	P-values
Spleen vs. TAb2	<0.0001
Spleen vs. TCh3	<0.0001
TAb2 vs. TCh3	<0.0001

CD206$^+$CD86$^+$	P-values
Spleen vs. TAb2	0.0099
Spleen vs. TCh3	0.8605
TAb2 vs. TCh3	0.0403

Figure 2G

IFNγ$^+$	P-values
SPLEEN (TCh3) vs. TAb2	<0.0001
SPLEEN (TCh3) vs. TCh3	<0.0001
TAb2 vs. TCh3	0.0115

TNFα$^+$	P-values
SPLEEN (TCh3) vs. TAb2	<0.0001
SPLEEN (TCh3) vs. TCh3	<0.0001
TAb2 vs. TCh3	0.8422

TNFα$^+$IFNγ$^+$	P-values
SPLEEN (TCh3) vs. TAb2	0.2604
SPLEEN (TCh3) vs. TCh3	0.0066
TAb2 vs. TCh3	<0.0001

B

Figure 8B

CD11b$^+$	P-values
aPDL1 vs. TCh3	>0.9999
aPDL1 vs. SP	0.0022
TCh3 vs. SP	<0.0001

CD4$^+$	P-values
aPDL1 vs. TCh3	>0.9999
aPDL1 vs. SP	0.0031
TCh3 vs. SP	0.0182

CD8$^+$	P-values
aPDL1 vs. TCh3	0.0058
aPDL1 vs. SP	0.6974
TCh3 vs. SP	<0.0001

IFNγ$^+$	P-values
aPDL1 vs. TCh3	0.0154
aPDL1 vs. SP	<0.0001
TCh3 vs. SP	<0.0001

TNFα$^+$	P-values
aPDL1 vs. TCh3	0.7611
aPDL1 vs. SP	<0.0001
TCh3 vs. SP	<0.0001

TNFα$^+$IFNγ$^+$	P-values
aPDL1 vs. TCh3	0.9737
aPDL1 vs. SP	0.0273
TCh3 vs. SP	0.0152

C

Figure S5A

F4/80$^+$ in CD11b	P-values
BM (media only) vs. TAb2 BM (media only)	<0.0001
BM (media only) vs. TCh3 BM (media only)	0.1320
TAb2 BM (media only) vs. TCh3 BM (media only)	<0.0001

Figure S5B

CD11b$^+$CD11c$^+$ in CD45	P-values
BM (media only) vs. TAb2 BM (media only)	0.6371
BM (media only) vs. TCh3 BM (media only)	0.0011
TAb2 BM (media only) vs. TCh3 BM (media only)	0.0084

Figure S5C

CD11b$^+$ in CD45	P-values
BM (media only) vs. TAb2 BM (media only)	<0.0001
BM (media only) vs. TCh3 BM (media only)	<0.0001
TAb2 BM (media only) vs. TCh3 BM (media only)	<0.0001

Figure S5D

M-MDSC in CD11b	P-values
BM (media only) vs. TAb2 BM (media only)	<0.0001
BM (media only) vs. TCh3 BM (media only)	<0.0001
TAb2 BM (media only) vs. TCh3 BM (media only)	0.9967

Figure S5E

PMN-MDSC in CD11b	P-values
BM (media only) vs. TAb2 BM (media only)	0.5874
BM (media only) vs. TCh3 BM (media only)	<0.0001
TAb2 BM (media only) vs. TCh3 BM (media only)	<0.0001

D

Figure S8A

CD11b$^+$	P-values
Spleen vs. TAb2	0.0004
Spleen vs. aPDL1	0.0014
TAb2 vs. aPDL1	>0.9999

CD4$^+$	P-values
Spleen vs. TAb2	<0.0001
Spleen vs. aPDL1	0.0045
TAb2 vs. aPDL1	0.7918

CD8$^+$	P-values
Spleen vs. TAb2	0.0002
Spleen vs. aPDL1	0.0021
TAb2 vs. aPDL1	>0.9999

Figure S8B

M-MDSC	P-values
TAb2 vs. aPDL1	0.7835
TAb2 vs. Spleen	0.8203
aPDL1 vs. Spleen	0.9974

PMN-MDSC	P-values
TAb2 vs. aPDL1	0.0701
TAb2 vs. Spleen	0.001
aPDL1 vs. Spleen	<0.0001

Figure S8C

F4/80	P-values
Spleen vs. TAb2	0.0059
Spleen vs. aPDL1	<0.0001
TAb2 vs. aPDL1	0.9439

Figure S8D

IFNγ$^+$	P-values
Spleen vs. TAb2	<0.0001
Spleen vs. aPDL1	<0.0001
TAb2 vs. aPDL1	0.9396

TNFα$^+$	P-values
Spleen vs. TAb2	<0.0001
Spleen vs. aPDL1	<0.0001
TAb2 vs. aPDL1	0.9858

TNFα$^+$IFNγ$^+$	P-values
Spleen vs. TAb2	0.0003
Spleen vs. aPDL1	0.0003
TAb2 vs. aPDL1	0.9932

Figure S9. Detailed P-values for figures. (A) Statistical significance was calculated using Kruskal-Wallis test for Figure 2A and Two-way ANOVA for Figures 2C, E and G. **(B)** Statistical significance was calculated for Figure 8B using Two-way ANOVA. **(C)** Statistical significance for Figure S5 Day 4 time point was calculated using Two-way ANOVA. **(D)** Statistical significance was calculated using Kruskal-Wallis test for Figure S8A and Two-way ANOVA for Figures S8B-D.

Figure S9
